# Study of Hemp Fiber Properties Modified via Long-Duration Low-Pressure Argon and Oxygen Plasma Treatments

**DOI:** 10.1021/acsomega.5c06420

**Published:** 2025-12-11

**Authors:** Kunal Bapat, Lekshmi Kailas, Christopher Pask, Terence P. Kee, Stephen Russell

**Affiliations:** † School of Chemistry, 4468 University of Leeds, Woodhouse Lane, Leeds LS2 9JT, U.K.; ‡ School of Physics and Astronomy, 4468 University of Leeds, Woodhouse Lane, Leeds LS2 9JT, U.K.; § Leeds Institute of Textiles and Colour, School of Design, University of Leeds, Woodhouse Lane, Leeds LS2 9JT, U.K.

## Abstract

Hemp is a lignocellulosic fiber used in fiber-reinforced composites, technical textiles, and clothing with surface properties that can be modified by plasma to improve processability. In contrast to previous studies reporting the effects of short-duration plasma treatment (<10 min), this paper investigates the effects of extended (30 min–4 h), low-pressure (∼0.4 mbar) argon and oxygen plasma treatments on dew retted hemp fibers at varying power levels (40 and 80 Hz). Scanning electron microscopy (SEM) revealed marked surface fiber etching after prolonged treatment, with argon plasma inducing fibrillation and heterogeneous motifs, while oxygen plasma yielded irregular morphologies. Atomic force microscopy (AFM) confirmed a near 4-fold rise in surface roughness (70 to 270 nm) after 4 h of plasma treatment. All plasma-treated fibers exhibited complete wetting (water contact angle θ = 0°) versus θ = 62° for untreated controls, based on drop-shape analysis and tensiometry. Fourier transform infrared spectroscopy (FT-IR) revealed no major chemical shifts, although sharper −OH and −C=O peaks suggested a subtle physicochemical change. X-ray diffraction indicated slightly enhanced crystallinity without crystallite size alteration. Fiber tensile strength remained unaffected across treatments. Fluorescence microscopy suggested a degree of lignin removal, evidenced by reduced surface fluorescence after 4 h of argon plasma treatment. Thus, long-duration argon and oxygen plasma treatments distinctly modify hemp fiber surfaces, without substantially altering internal chemistry or crystallinity. These findings highlight plasma treatment as an alternative to wet chemical methods for surficial hemp fiber modification, offering potential for precise surface engineering in textile applications.

## Graphical abstract

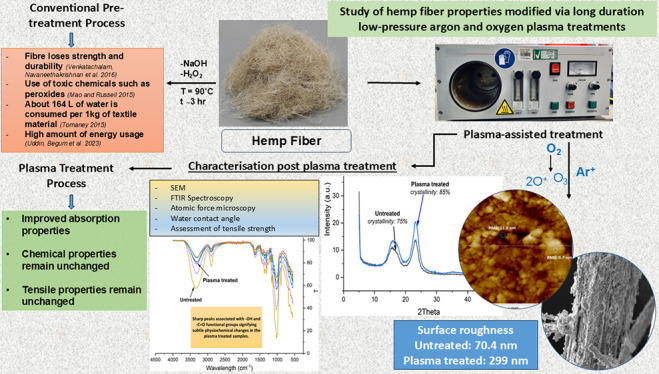

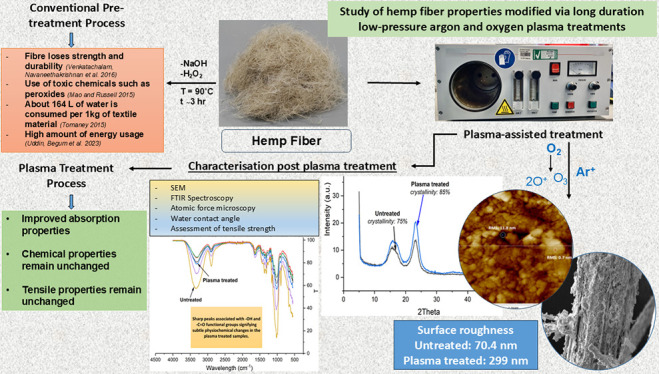

## 1 Introduction

Lignocellulosic bast fibers such as hemp are used to produce natural fiber-reinforced composites (FRCs), technical textiles including architectural and building products, as well as fabrics for garments.^1,2^ The attractive tensile strength (∼700–900 MPa),^3^ modulus (∼75 GPa),^3^ and economics of hemp fiber production have led to its resurgence in eco-conscious fashion and textile products, where the environmental impacts of fiber production, including resource extraction and emissions, are increasingly scrutinized. Of course, the precise environmental footprint and physical properties of hemp depend on the agricultural system and conditions used to grow the plant, as well as the fiber processing methods that follow.

To extract industrially valuable hemp fibers from plant stems for textile manufacturing, substantial preprocessing is required, including retting after harvesting. Dew retting, also referred to as field retting, is a cost-effective method where harvested hemp is left in the field for 3–5 weeks, allowing microbial activity to break down the pectin, hemicellulose, and lignin that binds the integral fibers. Decortication (separating the fibers from the woody core, or shiv), scutching (beating the fibers to remove any remaining woody material), and hackling (mechanical cleaning and fiber separation) are required to produce clean, high-quality fibers.^4,5^

Hemp fiber degumming is typically performed following retting, scutching, and hackling. Fibers obtained through dew retting may require intensive degumming due to residual pectin content.^6^ Consequently, the choice of degumming method is tailored to the intended end-use, with chemical and enzymatic approaches commonly employed to produce high-quality textile-grade fibers.^6^ Noncellulosic impurities, mainly lignin, pectin, and hemicellulose, can be removed through degumming^7^ typically using chemical (normally alkali),^8^ enzymatic, or eutectic solvent methods.^9,10^ After degumming, hemp fiber comprises mainly cellulose by mass (65%–75%), but residual impurities such as lignin are still present (∼9%) axially distributed within the fiber, along with hemicellulose (∼20%), pectin, and fats, which comprise 3% of the total. During subsequent processing, these impurities adversely affect surface properties, such as liquid wetting. However, while lignin removal improves wettability, it often compromises fiber strength, and significant variability in fiber length, diameter, and surface characteristics persists.^3,11,12^ Strategies for lignin removal include alkaline treatment, oxidative treatments, and enzymatic treatments, which can be harnessed to reduce fiber diameter. Cottonization further reduces the fiber diameter, enabling the spinning of fine yarns for textile fabrics suitable for clothing. For the cottonization of hemp fibers, various methods can be employed, including the use of deep eutectic solvents, urea-etidronic acid treatment, and soda-lye scouring.^13^ The combination of deep eutectic solvents and microwave energy enhances the removal of impurities and enables the efficient extraction of pure cellulose from hemp fibers. In contrast, soda-lye scouring not only refines the fibers but also imparts inherent flame-retardant properties.^14,15^ Deep eutectic solvent (DES) treatments can reduce fiber tensile strength due to the removal of noncellulosic components such as lignin and hemicellulose, potentially rendering the fibers more brittle.^16^ While DES processing can enhance specific properties desirable for textile applications, it may also compromise structural integrity and alter chemical composition. Therefore, careful optimization of treatment parameters is essential to balance performance gains with mechanical durability depending on the intended end use. These wet processes aim for 70–100% delignification, but the processes are associated with significant chemical effluent and resource consumption.^11^

Wet oxidation, hydrothermal treatment, and steam explosion can also increase the cellulose content in the fiber by up to 15%.^17^ Additionally, esterification, alkalization, silane treatments, and UV treatments have been identified as advantageous for improving the mechanical properties of hemp fiber.^3^ While aqueous pretreatment methods for hemp are highly effective in removing impurities and increasing the cellulose content, they utilize significant quantities of water, chemical reagents, and energy, having significant effluent management requirements. An alternative approach that obviates the need for wet chemistry and associated drying is attractive if valuable modifications to the fiber surface can still be obtained.

Previously, oxygen,^18^ atmospheric air,^19^ nitrogen,^20^ or argon^21^ gas plasma treatment of hemp has been reported as “dry” means for modifying fiber properties such as wetting, chemical functionalization, and surface cleaning.^22^ Hamad et al. treated ramie fibers with atmospheric air plasma for short durations ranging from 60 to 240 s, resulting in significant nanotexturing of the surface. This reduced the water contact angle from 50° to 30°, improving wettability, while the chemical functional groups of cellulose in the fibers remain unaffected.^23^ Another study by Pejić et al. examined the sorption properties of hemp fibers treated with atmospheric air plasma for 120 s at different power levels (40 and 80 W). A substantial increase in wettability was observed without any significant changes to the chemical or morphological properties of the fiber.^24^ A further study on the surface modification of 100% hemp woven fabrics using low-pressure argon plasma revealed nanoetching of fiber surfaces and enhanced wetting and dyeability, together with an increase in the number of polar groups after 10 min of treatment.^25^

Plasma treatment is widely recognized for its ability to achieve dry etching,^26^ resulting in nanoscale surface roughness that can substantially improve both the surface energy and the functional performance of natural fibers.^27^ The formation of roughened surfaces increases the available surface area, which in turn enhances interfacial adhesion in fiber-reinforced composites and strengthens interactions with finishing agents, dyes, and chemical coatings.^25^ One of the primary drawbacks of conventional wet chemical and enzymatic treatments is their tendency to compromise structural integrity, often resulting in a measurable loss of fiber strength and flexibility due to uncontrolled molecular degradation.^28^ Plasma processing offers a compelling alternative, enabling the achievement of surface modification without altering the bulk fiber properties. The outcome of surface modification via plasma treatment is also strongly influenced by the type of reactive gas used; for example, the use of hexafluoroethane gas as a plasma source leads to the generation of hydrophobic surfaces. In contrast, gases, such as argon and oxygen, offer pathways for increasing surface polarity and hydrophilicity. Traditional lignin-removal strategies, including scouring and bleaching, frequently reduce fiber strength as a result of cellulose chain cleavage. The current study demonstrates that extended plasma treatments, particularly with argon, can reduce surficial lignin while fully retaining the tensile strength of the fibers, positioning this method as a robust alternative to aggressive chemical pretreatments. Moreover, although argon and oxygen plasmas have been individually explored in previous work, no comprehensive comparison exists evaluating their effects under prolonged low-pressure conditions. This work fills that gap by providing a systematic side-by-side analysis of the modifications in surface morphology, surface chemistry, and wetting behavior of hemp fibers subjected to extended-duration treatments in both argon and oxygen plasma environments. A process can only be fully understood when it is studied across its full operational window, from short to long durations. This broad-spectrum approach not only clarifies the range of phenomenological outcomes but also lays the foundation for tailoring treatments for diverse end-use applications.

This work primarily aims to broaden the current scope of literature, which often limits hemp’s application to basic garment substrates. Rather than focusing on a single end-use such as dyeing or finishing, the central objective of this study is to explore and understand the underlying mechanism of plasma–fiber interactions. By investigating the morphological, chemical, and physical changes induced through plasma treatment, we present a foundational understanding that can support a wide range of textile applications in both apparel and technical textile domains.

In contrast to previous studies on hemp focusing on short oxygen and argon plasma treatment times (typically <10 min), the aim of the present study was to examine the effects of longer treatments (30 min to >4 h) on fiber surface morphology, surface wetting, crystal structure, and fiber tensile properties. The purpose was also to identify any changes that could enhance fiber compatibility with downstream textile product manufacturing.

## 2 Experimental Section

### 2.1 Materials

Raw field-retted hemp fibers were procured from East Yorkshire Hemp Ltd.^29^ in the UK. Following dew retting process lasting 6 weeks, a series of mechanical processing steps were conducted by the supplier to yield fiber, prior to baling. The mechanical processing involved shredders to break down hemp stalk into smaller pieces, a decorticator to separate the hemp fiber from the hemp shiv, and last, cleaning machinery to remove dust and other debris. Argon and oxygen gas cylinders (supplied by BOC Ltd.) were utilized for plasma treatment, and the gases were used without further purification.

### 2.2 Method for Processing Hemp Fibers before Plasma Treatment

Retted hemp fibers from the received bale (1 kg) were mechanically opened using a laboratory-scale Tatham Mini PO30 fiber opener, consisting of a nipped feed roller and single-cylinder opener roller, to separate the constituent fibers and remove residual shiv. The hemp fiber was fed through the fiber opening process twice to maximize fiber separation. The opened fiber was then carded using a lab-scale, single-cylinder, worker-stripper sample card (Haigh), enabling intensive fiber disentanglement prior to web formation and production of a self-supporting sample for plasma treatment. No additional scouring or chemical preprocessing of the baled fiber or sliver was performed to ensure the effects of plasma treatment could be satisfactorily detected.

### 2.3 Plasma Treatment of Hemp Sliver

Research-grade pure argon and oxygen gases were used as a plasma source for the treatment of hemp fiber samples. A Diener Zepto low-pressure plasma machine (Diener Electronic GmbH & Co KG, Germany) was employed for the plasma treatment. The machine is equipped with two needle valves for precise gas supply with gas flow controllers. Operating in manual mode, the system is attached with a Pfeiffer Duo 3 rotary-vane vacuum pump to create a low-pressure environment in the plasma chamber. Plasma treatment was conducted at two power levels (intensity of plasma frequency) of 80 and 40 Hz for treatment durations of *t* = 30, 60, 120, 180, and 240 min. The gas supply for both gases was maintained at a constant flow rate, and a fixed pressure of 1 bar, to maintain a stable pressure of 1.5 mbar within the plasma chamber. After plasma treatment, individual fibers (∼500 fibers) were taken from the plasma-treated sliver and were conditioned in a standard textile testing environment (ISO 139), with a relative humidity of 65% and a temperature of 20 °C, before physical property measurements and chemical analysis.

### 2.4 Fiber Morphology Analysis

#### 2.4.1 Scanning Electron Microscopy

The surface morphologies of both untreated and plasma-treated hemp fibers were examined by using scanning electron microscopy (SEM). Micrographs were captured using a Jeol JSM 6610 SEM operating in secondary electron mode with an accelerating voltage (electron source energy) of 5.00 kV, covering a range of magnifications from 25× to 4000×, with a scan size varying from 1 mm to 10 μm × 10 μm following the method referred to by Juhaśz et al.^30^ Before imaging, the fibers were sputter-coated with gold by using a LUXOR gold sputter-coating device to yield a coating thickness of 50 nm.

#### 2.4.2 Atomic Force Microscopy

The surface topography of hemp fibers was examined using atomic force microscopy (AFM) (Bruker Dimension FastScan) operating in the tapping mode in air. Individual fibers (*n* = 3 samples) with an approximate length of 8 mm were affixed to round stainless steel specimen discs of 15 mm diameter using double-sided adhesive tape to ensure stability and flatness before scanning. FastScan A probes (Bruker) with a nominal spring constant of 18 N/m and a resonant frequency of 1.4 MHz, having an approximate diameter of 10 nm, were used for imaging each specimen. To ensure the reproducibility of the acquired AFM scans, untreated hemp fibers were initially imaged under the AFM. Three different areas on three different fibers were imaged, and scan sizes at 5, 10, 20, and 30 mm were collected from each area using the NanoScope 9.4 software. Images were acquired at a 768 × 768 pixel resolution at a scan rate of 1 Hz. These same untreated, mounted fiber samples were then plasma-treated using the method outlined in Section 2.3. Following plasma treatment, the samples were reexamined under the same AFM settings, within the same areas initially imaged. The obtained topographic images were processed with first-order flattening and analyzed by using NanoScope Analysis 1.9 software.

### 2.5 Chemical Structure of Fibers

The crystallinity of untreated and plasma-treated hemp fibers was analyzed using a Bruker D2 Phaser equipped with a Cu (1.541 Å) source and a LYNXEYE detector. The analysis was conducted at a scan speed of 5° per min, with the hemp fibers placed in a 35 × 50 × 5 mm glass sample holder and performed under plateau conditions.^31^ The crystallinity index was measured based on the diffractometric method outlined by Chukhchin et al.^32,33^ Data analysis and peak deconvolutions of the X-ray diffraction pattern(s) were done by using the OriginPro software. Additionally, the chemical structure was explored using a PerkinElmer FT-IR Spectrum-3 instrument equipped with a single-bounce diamond attenuated total reflection (ATR) accessory. Spectroscopic data were collected at 4 cm^–1^ resolution over 100 scans for untreated and plasma-pretreated samples. OriginPro software was used for the data representation of the obtained FT-IR scans.

### 2.6 Fiber Surface Wetting Analysis

Surface wetting analysis was conducted on fibers by using a Kruss BP100 tensiometer. Prior to analysis, microscopic images with a magnification ranging from 1 to 200 mm were taken using a Leica digital microscope, and ImageJ software was employed to measure fiber diameters. To reduce experimental error due to the inherent variation in the diameter of hemp fibers, specimens of fiber were selected with a diameter of 50 mm and cut into 10 cm lengths. The measurements involved assessing the force of attraction of solvents by varying the position of the fiber immersion. Hexane, an aprotic solvent, and water, a protic solvent, were utilized for this purpose.^34^ The detection speed for the tensiometer was set at 6 mm/min with a detection sensitivity of 5 × 1 × 10^–4^ mN/m. The measuring speed was kept at 3 mm/min with a maximum immersion depth of 5 mm and a minimum depth of immersion of 1 mm. The water contact angle was determined using the data acquired from the tensiometer.^35^ In the tensiometer setup, the fibers are suspended from the tip, allowing for measurement of both the force with which the fibers interact with water and the depth to which they are immersed. The Wilhelmy plate method was applied to determine the water contact angle.^36^ In the case of natural fibers such as hemp, which exhibit significant variation in diameter, ensuring the reproducibility of results is crucial. Therefore, the water contact angle was also measured using sessile drop measurements with a Kruss DSA30E goniometer.^25^ The water contact angle was measured by suspending a 7 mL sessile water droplet of deionized water with a surface tension of 72 N/m on the surface of plasma-treated and untreated lignocellulosic hemp fiber. The droplets were allowed to stabilize for 20 s, and the contact angle was measured using a built-in camera. From the recorded video, a series of images enabled the water contact angle to be measured at the point of the droplet’s contact with the surface. Three repetitions were performed for each fiber sample.

### 2.7 Fiber Tensile Properties

The tensile strength of both untreated and plasma-treated hemp fibers was evaluated using an Instron 5544 universal tensile strength tester following the BS EN ISO 5079-2020 test standard method for fiber testing.^37^ The crosshead speed was maintained at 20 mm/min using a gauge length of 20 mm.^38^ Before testing, the fibers were mounted on a 30 × 30 mm (length × breadth) standard template, which was cut before initiating the test. To ensure consistency, 15 replicates were tested, and the data were averaged. The tensile strength data was analyzed statistically using response surface methodology with the help of Minitab software. To minimize the degree of variation, lignocellulosic hemp fibers possessing a 50 μm diameter were selected for testing. Images of the fibers were captured by using a Leica optical microscope and analyzed with ImageJ software to measure the fiber diameter. Fiber testing was conducted in a controlled textile testing environment maintained at 65% relative humidity and 20 °C temperature. To ensure equilibrium with the testing conditions, the fibers were conditioned in this environment for 48 h before testing.

### 2.8 Fluorescence Microscopy

Fluorescence microscopy was employed to visualize lignin within hemp fibers by exploiting its inherent autofluorescence.^39^ Comparative analysis was conducted between untreated fibers and those subjected to argon plasma treatment for 4 h at 80 Hz, focusing on potential changes in lignin distribution and relative abundance. Fibers with diameters of 60–80 μm were selected to ensure morphological consistency. High-resolution imaging was performed using a Stellaris 8 confocal microscope, capturing Z-stack images to obtain three-dimensional optical sections of the fiber. Scanning was conducted at a rate of 10 μm/min to ensure precise depth profiling. Lignin fluorescence was excited using dual-wavelength illumination at 488 nm and 550 nm,^40^ delivered at 10% transmission with 30% laser power, parameters optimized to enhance signal intensity while minimizing photobleaching. Emission was detected using a band-pass filter (BP530) and a long-pass filter (LP590), allowing selective capture of lignin-specific signals in the green-to-red spectrum. This methodology enabled clear and specific visualization of lignin distribution in alignment with established excitation–emission parameters for lignified plant tissues.

## 3 Results and Discussion

As-received untreated (dew retted hemp fiber) and plasma-treated hemp fibers were examined to explore any differences in physical, morphological, and chemical properties.

### 3.1 Morphological Analysis of Plasma-Treated and Untreated Hemp Fibers

The SEM and AFM scans (Figure 1) on untreated and argon plasma-treated hemp fibers (40 Hz power for 30 min and 80 Hz power for 30 min) reveal clear differences in surface morphology. Comparing the SEM images (Figure 1A,D,G), it is evident that as plasma treatment time and the intensity of argon plasma increase, the degree of surface etching increases, with the maximum surface roughness determined by AFM increasing from approximately 530 nm to around 1.3 μm (Figure 1C,F,I). The surface morphology of the untreated fiber (Figure 1A) appears comparatively smooth, while argon plasma treatment for 30 min at 40 and 80 Hz power (Figure 1D,G) roughens the surface. Morphological distinctions are also visible in fibers treated with argon gas plasma after 30 min when altering the plasma power from 40 to 80 Hz (Figure 1D,G). Specifically, in the case of D, there appears to be a pronounced surface roughening, most probably caused by the removal of surficial layers associated with the multiple impacts of highly reactive ions, electrons, and radicals within the plasma.^41^ Sample G, which was treated at 80 Hz for 30 min, also showed evidence of the initiation of fibrillation. Fibrillation appears to be linked to surface roughness, which leads to the breakdown of the internal structure within the surface layers. This breakdown, however, is not anticipated to be thermally induced, as low-pressure plasma devices operate at low temperatures.^42^

**Figure 1:**
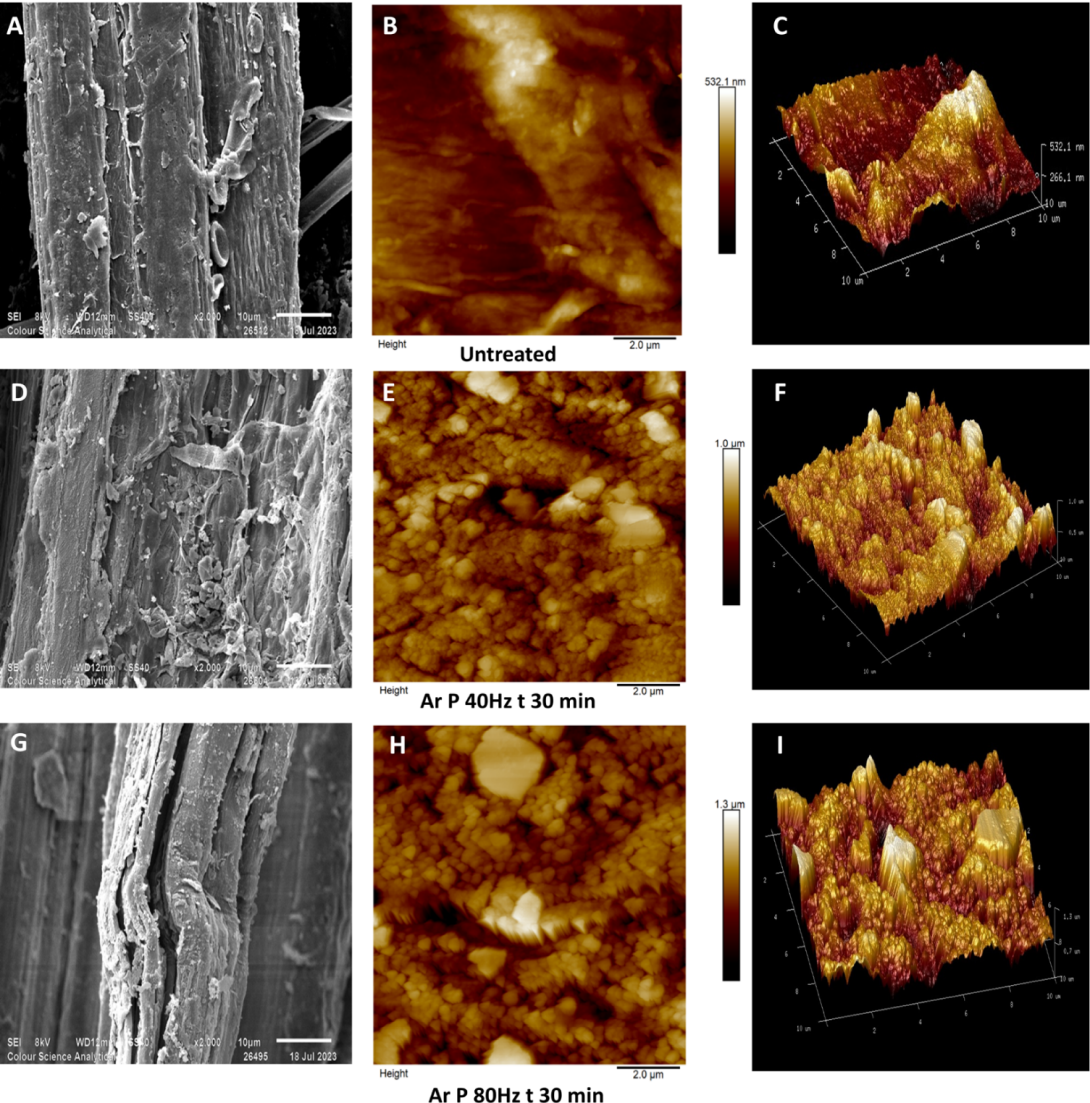
Scanning electron micrographs (×2000) and AFM (10 μm × 10 μm) scans of untreated and argon plasma-treated hemp fibers. The SEM micrographs in figures are taken at 2000× magnification, while the AFM scans cover a 10 μm × 10 μm area along with the 3D surface topography [A–C: SEM and AFM micrographs of untreated fiber with their 3D projection, D–F: SEM and AFM micrographs of fibers argon plasma-treated for 30 min at 40 Hz power with their 3D projection, and G–I: SEM and AFM micrographs of fibers argon plasma-treated for 30 min at 80 Hz power with their 3D projection].

With reference to Figure 2A,D, the argon plasma components (consisting of highly energetic atoms and positively charged ions) collide with the lignin-rich fiber surface over a period of 4 h, resulting in a highly etched and textured surface with lignin and hemicellulose remnants clinging to the fiber surface. Hemp fibers are relatively coarse in terms of diameter, with lignin strongly bonded throughout their length,^43^ so it may be hypothesized that plasma treatment facilitates disruption of lignin-cellulose binding interactions, resulting in a morphologically heterogeneous, etched surface being observed. In the research by Ivanovska et al., similar results were observed when jute fibers were plasma-treated in the presence of atmospheric-pressure air plasma.^44^

**Figure 2:**
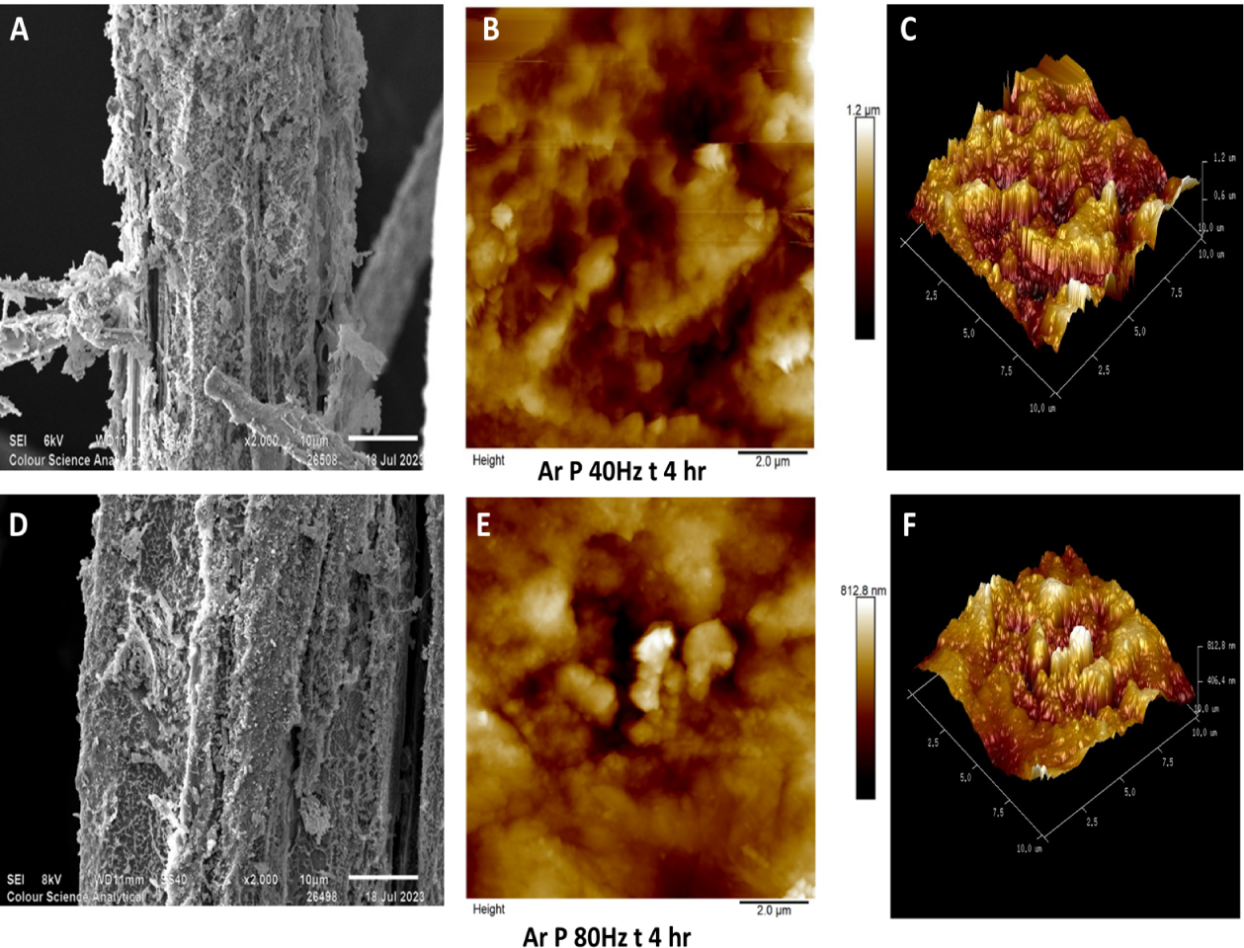
Scanning electron micrographs (×2000) and AFM (10 μm × 10 μm) scans of argon plasma-treated hemp fibers for a duration of four h at 80 and 40 Hz power [A–C: SEM and AFM micrographs of fibers argon plasma-treated for 4 h at 40 Hz power with their 3D projection and D–F: SEM and AFM micrographs of fibers argon plasma-treated for 4 h at 80 Hz power with their 3D projection].

The AFM topographical images over a scan length of 10 μm × 10 μm are given in Figures 1B,E,H, with their 3D surface projections in Figure 1C,F,I (untreated and argon plasma-treated hemp fibers for 30 min at 40 and 80 Hz). Additionally, AFM images in Figure 2B,E show hemp fiber samples plasma-treated for a substantially longer period of 4 h at 40 and 80 Hz power, respectively. The AFM scans of untreated fibers (Figure 1B,C) reveal a relatively smooth surface, with negligible peaks and troughs on the surface, whereas argon plasma-treated fibers exhibit a roughened surface texture across the full 10 μm × 10 μm scan and are particularly noticeable in fibers plasma-treated for a shorter treatment time of 30 min (Figure 1E,H). Their 3D surface projection, illustrated in Figure 1F,I, clearly shows a highly textured surface. These topologically altered features on the fiber surface may be attributed to lignin.^41^ Previous AFM studies conducted on pretreated eucalyptus wood reported similar results to those reported here, albeit with a different material.^45^ The textured surface could also be associated with physical disruption of the surface, including fibrillation.^46^ Increasing the treatment time to 4 h led to the formation of deeper valleys, and cracks became more pronounced (Figure 2B,C,E,F).

In Figure 3, SEM images, taken at 1200× magnification, alongside 10 μm × 10 μm AFM scans, illustrate the surface morphology and topography of hemp fibers treated with oxygen gas plasma under different conditions. Fibers treated at 40 Hz for 30 min (Figure 3A) show a slightly rougher surface than untreated fiber samples, while those treated at 80 Hz for the same duration (Figure 3D) display more pronounced surface etching of the fiber surface. Fibers treated for 4 h at 40 Hz (Figure 3G) exhibit a highly roughened, network-like surface structure. de Farias et al.’s work on oxygen plasma-treated coir fibers showed a similar network-like structural morphology.^47^

**Figure 3:**
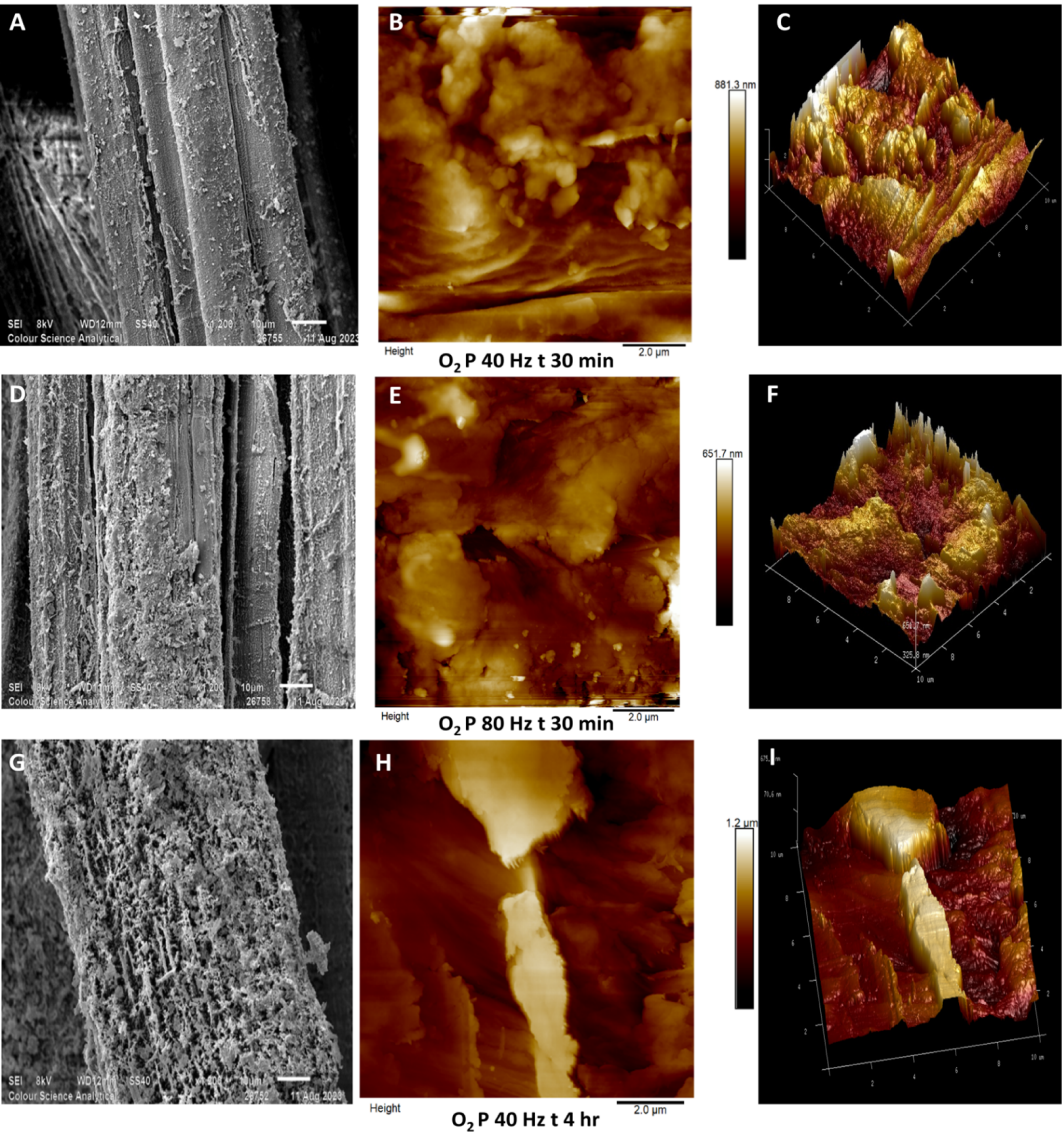
Scanning electron micrographs (×1200) and AFM (10 mm × 10 μm) scans of untreated hemp fibers and pretreated fibers using oxygen gas plasma [A–C: SEM and AFM micrographs of fibers oxygen plasma-treated for 30 min at 40 Hz power with their 3D projection, D–F: SEM and AFM micrographs of fibers oxygen plasma-treated for 30 min at 80 Hz power with their 3D projection, and G–I: SEM and AFM micrographs of fibers oxygen plasma-treated for 4 h at 40 Hz power with their 3D projection].

Subsequent AFM scans (10 μm × 10 μm) of fibers plasma-treated with oxygen gas are illustrated in Figures 3B,E,H with their 3D surface topography in Figure 3C,F,I. Note that the roughened surface globular features that were observed in argon plasma-treated fibers are less visible in these AFM scans. Lignin consists of complex macromolecular aggregates,^48^ because of which it can be observed in globular structures similar to those prominently seen in Figures 1E,H. Micic’s AFM work on lignin shows similar globular structures.^49^ Comparing AFM scans in Figures 1E,H, 2B,E and 3B,E,H), it may be speculated that oxygen plasma is more capable of lignin removal than argon plasma. Muazzam et al. examined lignin following oxygen plasma treatment and found that lignin underwent oxidation due to the presence of ozone in oxygen plasma.^50^

In a study by Edwards et al., the regenerated cellulose fiber lyocell was plasma-treated for 20 min at 40 Hz power. AFM studies revealed a change in surface roughness from 0.18 nm, in the case of untreated lyocell, to 3.18 nm after oxygen plasma treatment.^51^ Chemically, lyocell contains negligible impurities in the form of lignin, hemicellulose, and pectin, comprising up to 92% α-cellulose.^52^ The marked changes in the surface morphology of hemp fibers observed herein are therefore suggested to be consistent with the removal of impurities such as lignin, hemicellulose, and pectin, as well as the sputtering effects during plasma treatment.

Oxygen plasma primarily triggers chemical reactions with O_2_ gas, generating a plasma that is rich in reactive oxygen atoms and ozone (among others), which contribute to surface modification and surface functionalization through chemical transformation.^53^ In contrast, argon plasma consists of high-velocity Ar^+^ ions that modify the surface through physical mechanisms. The resulting differences in surface morphology arise from these distinct processes of oxygen plasma, causing chemical changes, and argon plasma, inducing physical alterations. Yamamoto et al. studied the difference in argon and oxygen plasma treatments on gold films and concluded that argon plasma had a physical effect and oxygen plasma had a chemical effect, the latter inducing the formation of Au_2_O_3_.^54^

Hemp fibers plasma-treated using oxygen gas possess a high number of cracks and valleys on the surface, and a prominent difference can be seen in samples treated for 30 min at 40 Hz (Figure 3B) and at 80 Hz (Figure 3E). Some features are indicative of globular lignin (Figure 3B), whereas increasing plasma power roughens the surface with deep valleys, hundreds of nanometers in depth (Figure 3E). A more substantial change is visible in fibers plasma-treated for 4 h at 40 Hz (Figure 3H) with an etched surface across the area, resulting in deeper cracks on the surface extending to more than 1 μm in depth, as indicated in the 3D surface projection (Figure 3I).

When comparing the effects of argon and oxygen plasma treatment on hemp fibers, it is evident that oxygen plasma produces eroded surface features^55^ on the fibers. It can be hypothesized that this is likely due to the presence of oxidizing components present in oxygen plasma,^56^ resulting in more aggressive chemical interactions than in argon plasma. In contrast, argon plasma primarily roughens the surface through an atomic-layer etching process,^57^ and herein, it can be said that present surface components such as lignin, hemicellulose, and pectin are removed one atomic or molecular level at a time,^25,57^ exposing textured features.^58^ Similar observations can be made referring to the section profile graphs in the Supporting Information (Figures S1–S3) determined for a 10 nm section in the AFM scan. These graphs with their marked sections on the AFM scans are illustrated in the Supporting Information.

In the AFM images, lighter colors indicate raised surface features, while darker colors represent recessed or engraved areas. By comparing AFM height images and 3D surface projections across Figures 1–3, it can be hypothesized that argon plasma treatment initially removes surface layers up to a certain threshold, after which a smoother surface with reduced roughness is achieved. It can be said that the action of physical sputtering due to the presence of Ar^+^ in argon plasma continues only until the reactive material (lignin, hemicellulose, or pectin) is fully removed. This type of threshold effect can be hypothesized and will be studied in the future. This is evident in Figure 2B, where the surface roughness measures ∼170 nm, although the surface roughness of argon plasma-treated hemp fiber (Figure 1B) is approximately double that value, suggesting that the treatment effectively levels out the surface after removal of the top layers. In contrast, oxygen plasma-treated hemp fibers possess a nonuniform, roughened surface topography with deep troughs (with a maximum depth of ∼1.2 mm) and peaks.

Plasma is known to be capable of both dry etching and nanotexturing polymer surfaces.^59^ This is also observed in argon and oxygen plasma-treated hemp fibers. In a recent study, lignin mapping was conducted on lignocellulosic nanofibers using AFM, revealing that lignin covers thin cellulose fibers and presents a grain-like structure.^60^ Additionally, AFM analysis on kapok fiber demonstrated similar bundle-like formations with surface roughness being modified by approximately 80% after plasma treatment.^61^ In the present study, AFM surface roughness data for both untreated and plasma-treated hemp fibers were analyzed (Table 1). Each roughness measurement is calculated as the average from three AFM scans of 10 μm × 10 μm scan. Referring to Table 1, untreated fibers were found to have a surface roughness of around 70 nm, i.e., the surface height range between the highest and lowest topographical points in the imaged area. This increased to ∼300 nm following argon plasma treatment at 40 Hz and 30 min duration.

**Table 1:** Surface Roughness of Untreated and Plasma Pretreated Hemp Fibers Measured Using the Root-Mean-Square (RMS) Method

Plasma treatment				
Power (Hz)	Duration (min)	Source gas	Scan size (μm)	Surface roughness (nm)
0	0	n/a	10	70.4
40	30	argon	10	299
80	30	argon	10	199
40	30	oxygen	10	101
80	30	oxygen	10	182
40	240	argon	10	105
80	240	argon	10	172
40	240	oxygen	10	111

Figures 1–3 suggest that plasma treatment introduces a nanotextured effect on the fiber surface. The surface roughness values in Table 1 are the root-mean-square values (RMS). The fiber treated with oxygen gas at 80 Hz power for a treatment time of 30 min produced a surface roughness of 182 nm for a 10 mm × 10 mm scan. Interestingly, the fiber sample treated for 4 h at 40 Hz power using oxygen gas measured a surface roughness of 111 nm in a 10 mm × 10 mm scan. To gain detailed insights into the surface topography, we also used “section analysis” to study the surface. Figures S1–S3 in the Supporting Information represent a section profile (denoted as a white line on the scan) and its surface profile graph. The most heightened point and the most depressed point are also marked. Surface response analysis and principal component analysis (multivariate analysis) of these three variables (power, treatment duration, and gas) as contributors to surface roughness were carried out and are illustrated in Figure S4 of the Supporting Information. Therefore, the statistical analysis suggests that plasma treatment operates as a synergistic process, where gas type, power, and treatment duration collectively influence surface modification. While each factor holds individual significance, it is the optimal combination of all three parameters that yields the most effective surface roughness outcomes.

The ablation observed in the fibers due to the oxygen gas plasma may be attributed to radical formation.^62^ When oxygen gas (O_2_) plasma is generated, electrons having a threshold energy of approximately 16.4 eV are produced along with additional metastable states, and the total energy of O_2_ plasma tends to increase.^26^ This causes an increase in the density of electronegative species, due to which the etching on the surface can be observed.^63^ Bès et al. studied the mechanism of oxygen plasma etching on hydrocarbons, concluding that the ablation of surfaces in the presence of oxygen gas plasma is due to the adsorption and desorption of energy-rich species present in the plasma.^64,65^ When oxygen (O_2_) gas contacts high-voltage electrodes, the electrical discharges break the molecule into oxygen atoms or ionize the molecule to form 2O^+^ ions. Additionally, oxygen atoms react with oxygen molecules to form O_3_, ozone, which is highly reactive and can contribute to dry etching.^66,67^ This complex interaction of plasma species (mixture of reactive components) leads to the observed nanotextured surface modifications in oxygen plasma-treated fibers, resulting in a surface having a roughness of 182 nm. Cao et al. studied plasma-lignocellulose interaction, wherein ball-milled wood was plasma-treated under atmospheric air. The study concludes that the degradation of plasma-treated lignin is mainly due to the cleavage of ether bonds present in lignin.^68^

Structurally, hemp fibers have an outer coating of lignin, with the studied fibers containing approximately 9% lignin, which is noncrystalline and amorphous, being hydrophobic.^69^ The etchant gas, either oxygen or argon, when in contact with high-voltage electrodes, generates electrons due to ionization. These electrons are generated rapidly due to frequent collisions, and the electron density in the plasma reactor increases exponentially.^70^ These electrons possess high temperatures and have an energy of up to 25 eV. Hence, it can be hypothesized that when these high-energy electrons and other ionized species encounter a relatively weak-bonded amorphous structure such as lignin bundles, the surface layer of lignin can be readily removed by such energetic impacting, leading to a distinctly etched surface.^71^ For fibers pretreated using argon gas, the surface roughness increased significantly from 70 to 299 nm. Argon gas is an inert gas, but when used for plasma generation, it results in the production of Ar^+^ along with electrons. Ar^+^ ion has a velocity of 800 m/s, and when it collides with the lignocellulosic fiber surface results in an etched surface appearing as nanotextured.^58^ Etching is a layer-by-layer process, and progressive etching can be observed as the treatment time increases from 30 min up to 4 h. The etching behavior of Ar and O_2_ plasmas on hemp fibers varies due to their distinct energy distribution profiles. Argon (having a relative mass of ∼40 u) delivers high-momentum physical sputtering, increasing surface roughness, while O_2_ plasma generates lighter atomic oxygen (with a relative mass of ∼16 u), enabling frequent collisions and chemical oxidation of lignin and hemicellulose. Thus, argon plasma can favor mechanical ablation, whereas oxygen plasma may react with a combined physical and chemical etching, and the detailed type of etching can be detected using chemical analysis like FTIR.

An increase in surface roughness can result in enhanced wetting and an increase in surface energy.^72^ Surface morphology plays a significant role in determining wetting characteristics, largely due to its influence on surface free energy, which is a key factor in wetting behavior. This relationship is traditionally explained by Young’s equation,^73,74^ which assumes ideal smooth surfaces. However, after plasma treatment, when a smooth surface (∼70 nm surface roughness) becomes highly roughened (∼150 to 200 nm), the modified Cassie–Baxter theory^75^ characterizes the wetting behavior more accurately. By considering all three theoretical models, Young’s model, Wenzel’s equation, and the Cassie–Baxter theory, it becomes evident that surface roughness is a crucial factor in controlling wetting behavior.^76^ This is because roughness affects the balance of free energies between the solid–vapor and solid–liquid phases, thus altering the overall wetting properties of the material. Consequently, the modification in the wettability of plasma-treated hemp fibers was studied.

### 3.2 Influence of Plasma Treatment on the Surface Wetting Property of Hemp Fibers

The wettability of hemp fibers having undergone oxygen and argon plasma treatment for 30 min to 4 h at 40 and 80 Hz power was determined by tensiometry.^77^ Advancing and receding contact angles were measured using distilled water and *n*-hexane as probe liquids. When *n*-hexane was used as a solvent, all of the plasma-treated samples, along with the untreated fiber sample, measured a water contact angle of 0°. This is because *n*-hexane is a nonpolar solvent with a surface tension of 18.4 mN/m. In contrast, water, being a polar solvent, has a surface tension of 72 mN/m.^78^ For the untreated fiber samples, the measured water contact angle was 65.2°, indicating that hemp fiber before plasma treatment is partly hydrophilic. After plasma treatment (Ar and O_2_) was carried out, the water contact angle reduced to 0°. This modification from a partly hydrophobic surface to a fully wettable surface is arguably due to changes occurring in the combined morphological–chemical alterations, leading to an increase in surface energy.

These results were confirmed through water contact angle measurements using a Krüss goniometer. The water contact angle for the untreated fiber sample was recorded at ∼69°, and all of the plasma-treated fiber samples showed a reading of 0°. The surface wetting of a fiber is influenced by factors such as surface roughness and chemical composition.^79^ For pure cellulose, the water contact angle typically ranges from 20° to 30°.^80^ Interestingly, the experimental data show that 100% wetting was achieved after plasma treatment by both gases, which may suggest that plasma treatment enhances the surface energy of the fiber.^44^ Herein, the surface roughness changed from 70 to 299 nm (Table 1). These experimental results align with the Wenzel model,^76^ and a water contact angle of 0° suggests that the surface energy of the fiber is enhanced due to both physical as well as chemical interaction of argon and oxygen plasma with the hemp fiber.^51^

This synergistic improvement in surface texture and wettability offers several functional advantages. The roughened nanoscale topography enhances the total surface area, promoting the capillary-driven adsorption and absorption of dyes, textile finishes, and functional coatings. Simultaneously, the elevated surface energy resulting from plasma-induced chemical alterations facilitates stronger molecular interactions and bonding with these agents. Such conditions are highly favorable for applications requiring efficient wet finishing, dyeing, or biofunctionalization. To further explore the chemical changes associated with plasma treatment, Fourier transform infrared spectroscopy (FTIR) and X-ray diffraction (XRD) analyses were conducted. These techniques provide insight into alterations in chemical bonding and crystalline structure, complementing the surface wetting observations and helping to elucidate the underlying plasma-induced modifications.

### 3.3 Influence of Plasma Treatment on the Chemical Structure of Hemp

FTIR spectra of untreated and plasma-treated hemp fibers are shown in Figure 4 (oxygen plasma) and Figure 5 (argon plasma), in which prominent peaks for −OH, −CH, and −C–O functional groups can be prominently observed in the frequency range from 4000 cm^–1^ to 800 cm^–1^. Referring to Figure 5, the untreated fibers show characteristic peaks at ∼3400 cm^–1^ (O–H stretching in cellulose and hemicellulose), ∼2900 cm^–1^ (C–H stretching in cellulose), and ∼1050 cm^–1^ (C–O stretching in cellulose). After oxygen plasma treatment, the spectra in Figure 4 showed sharp −OH and C–O stretching peaks at ∼3400 cm^–1^ and ∼1050 cm^–1^ wavelengths. Similarly, FTIR spectra of argon plasma-treated fibers in Figure 5 indicate an increase in the intensity of the −OH and C=O functional groups. Although FTIR probes both surface and near-surface regions, having a penetration depth of ∼1–5 μm, the observed spectral shifts in plasma-treated fibers indicate an enhanced fiber polarity.^81^

**Figure 4:**
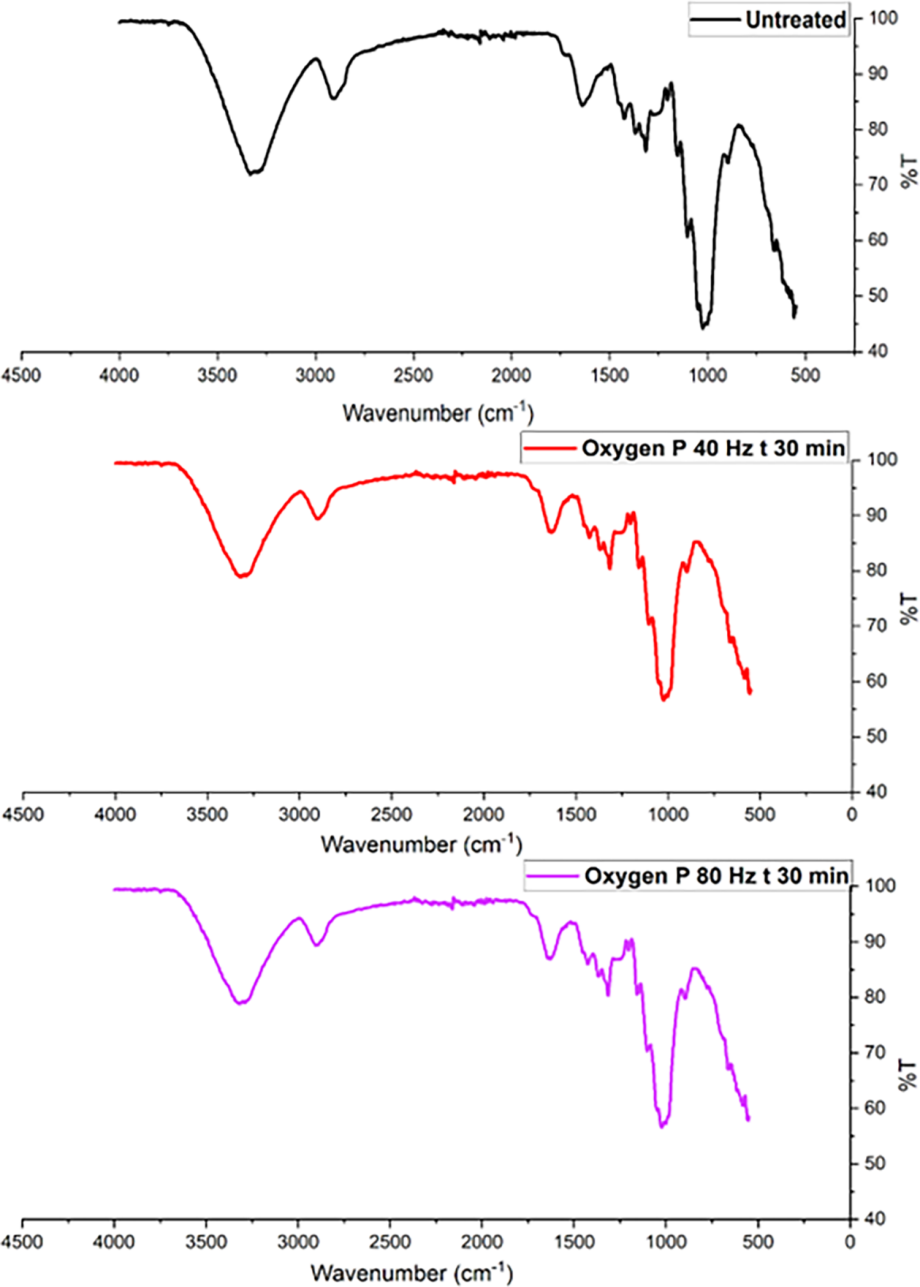
FTIR spectrum of hemp fibers before and after oxygen plasma treatment for 30 min with 80 and 40 Hz power.

**Figure 5:**
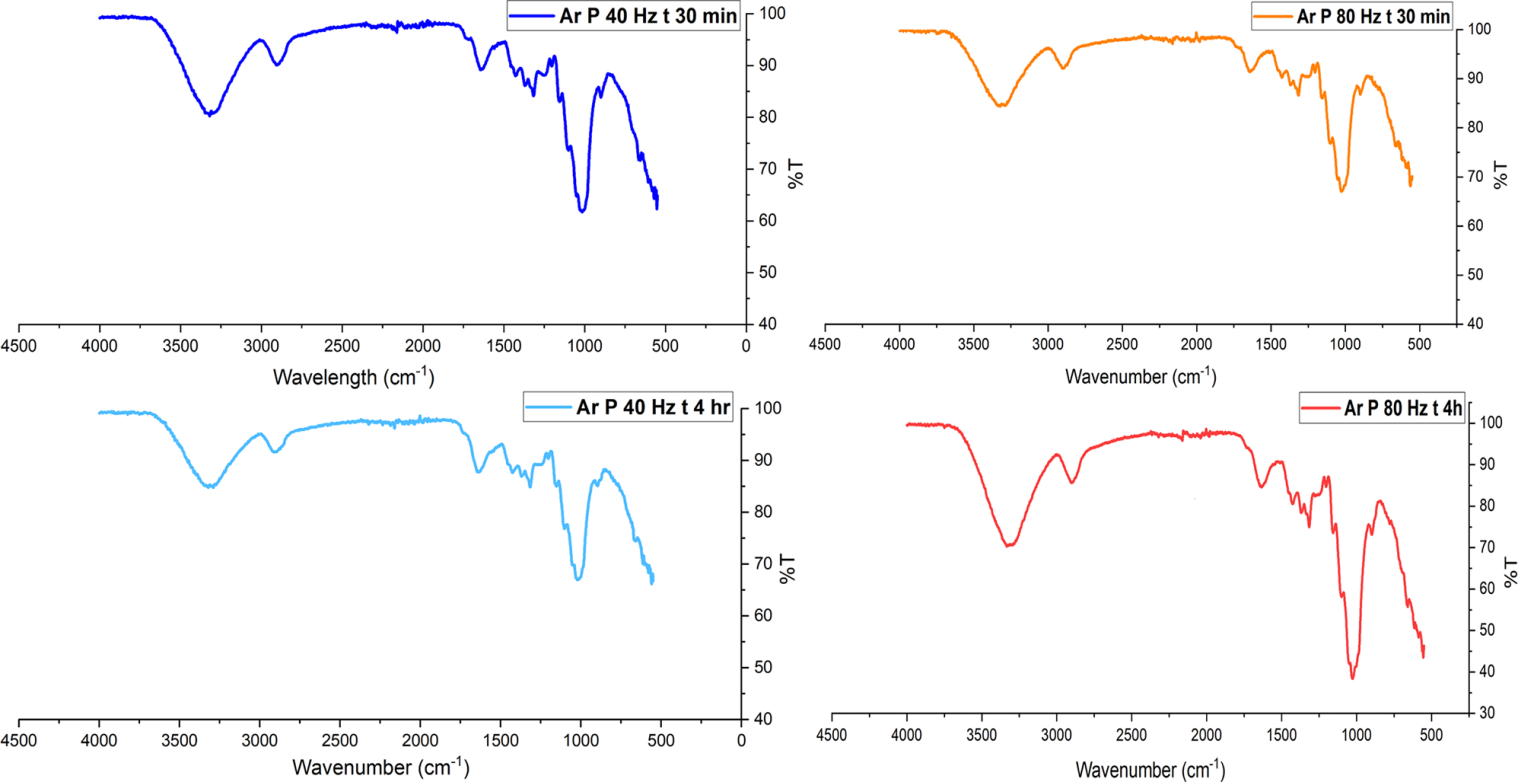
FTIR spectrum of hemp fibers before and after argon plasma treatment.

In FTIR analysis, the peak height (intensity) of a vibrational band is proportional to the concentration of the corresponding functional group as per Beer–Lambert’s law.^81^ Broad peaks suggest heterogeneity in the molecular environment, often due to interactions like hydrogen bonding, van der Waals forces, or dipole–dipole interactions between functional groups, and sharp peaks indicate more uniform environments.^81^ Following oxygen or argon plasma treatment, hemp fibers exhibit sharp peaks within the frequency range of 3500 cm^–1^ to 3000 cm^–1^, indicating two possibilities. The first possibility is surface activation, wherein the number of reactive functional groups (such as −OH) on the cellulose monolayer may increase, leading to sharper peaks.^54^ Another possibility is that plasma treatment may be linked to the surficial removal of lignin, hemicellulose, or pectin, resulting in changes in FTIR spectra of plasma-treated fibers showing sharper −OH peaks compared with the untreated fiber.^54^ In a recent study undertaken by Kostryukov et al., plant materials such as hemicellulose, lignin, and cellulose were analyzed using FTIR spectroscopy. FTIR analysis of extracted hemicellulose showed broader −OH peaks than the FTIR scan of cellulose or lignin, which showed sharp peaks for the −OH group.^82^

Regarding oxygen plasma treatment contributing to the functionalization of the substrate, these results do not show any alteration in the chemical fingerprint of cellulose. The sharp peaks at 3500 cm^–1^, 1300 cm^–1^, and 1100 cm^–1^ are evident after an extended duration of plasma treatment (30 min), which may lead to the breaking of weak surficial hydrogen bonds. A potential reason for the appearance of sharp peaks is the ring-opening of the glucopyranose units in the cellulose structure via a pyranosidic ring (C–O–C bonds) splitting mechanism.^83^ Although such a ring-opening reaction may be possible in the case of pure cellulose, it is rare for lignocellulosic hemp fibers.^56^

Figure 5 displays the FTIR spectra for both untreated and argon plasma-treated hemp fibers, revealing noticeably sharper peaks in the −OH fingerprint region (3500 to 3000 cm^– 1^). This suggests that exposure to argon plasma may lead to surface ablation, resulting in sharper peaks. A recent study by Kolářová et al. observed cellulose fibers extracted from cotton subjected to argon plasma for durations between 10 and 300 s, finding no evidence of dehydration, as indicated by the consistent −OH peak height.^84^ Similarly, Sawangrat et al. investigated chemical changes in bamboo fibers following argon and oxygen plasma treatments and found no notable alterations in functional groups, as the FTIR spectra for untreated and treated bamboo fibers showed minimal changes.^21^ In a recent study, argon plasma-treated hemp fabric produced similar findings, with the introduction of polar groups on the cellulosic surface evidenced by the appearance of sharper −OH.^25^

Non-pretreated hemp fibers display a yellowish hue due to the presence of lignin, with polyphenolic components such as syringyl and guaiacyl being detectable using FTIR spectroscopy.^85^ These lignin components display sharp peaks within the wavelength range of 1200 cm^–1^ to 1400 cm^–1^, belonging to functional groups such as C–C, C–H, and C–O.^86^ Hence, the use of FTIR as a primary method for the identification of pretreatment of lignocellulosic fibers is challenging, although sharp peaks associated with these functional groups indicate a physicochemical change in the substrate. In a study by Kabir et al.,^87^ the effects of alkalization on hemp fibers were examined. Higher concentrations of NaOH in the chemical treatment led to increased cellulose content in the hemp fibers, thereby eliminating lignin and hemicellulose^87^ and resulting in sharp −OH, −C–H, and −C–O peaks.^88^ Similar sharp peaks can be observed in the plasma-treated hemp fibers illustrated in Figures 4 and 5, suggesting that the functional groups of cellulose remain unchanged despite the physicochemical modifications. These changes result in more pronounced peaks, particularly in the case of the −OH group, indicating that plasma treatment enhances the visibility of these functional groups without altering their chemical structure.^25^

#### 3.3.1 Principal Component Analysis of FTIR Spectra

Table 2 presents the scores of different samples on the first two principal components (PC1 and PC2) obtained by a principal component analysis (PCA). PC1 has a variance of 98.2%, while PC2 has a 1.5% variance. The “Outlier” column indicates whether a sample is considered an outlier based on its position in the PCA space. The PC1 scores show a wide range of values, from −313.04941 to 641.22846. This suggests that PC1 captures a significant amount of variability between the samples. The PC2 scores have a smaller range compared with PC1, indicating that PC2 captures less variability. Two samples, which are outliers, were both plasma-treated with argon gas with the same power value of 80 Hz, although differing in the treatment duration, one for 30 min and the other for 4 h. This suggests that these samples have spectral characteristics distinct from the other samples. The wide range of PC1 scores indicates that the samples exhibit substantial differences in their spectral profiles.

**Table 2:** Percentage Contribution of Each Treatment in PC1 and PC2

Spectral Names	PC1 (98.2%)	PC2 (1.5%)	Outlier
Ar P40 t30	–188.54242	–23.13473	-
Ar P80 t30	–313.04941	–46.9618	Ar P80 t30
Oxygen P40 t30	–84.72997	31.09819	-
Ar P40 t4h	–274.64974	13.21125	-
Ar P80 t4h	219.74307	69.59966	Ar P80 t4h
Untreated	641.22846	–43.81257	Untreated

Figure 6A shows a loading plot, which is a technique commonly used in the principal component analysis (PCA). It displays the loadings of different variables (in this case, frequencies) onto the principal components (PCs). Loadings represent the correlation or weight of each variable in contributing to the variance explained by a particular PC. The bottom plot shows the loading of each frequency on the original data (Ar P40 t30). The middle plot displays the loadings of frequencies on the first principal component (PC1). PC1 explains 98.2% of the total variance in the data. Higher loadings indicate that a particular frequency contributes more to the variance captured by PC1. The top plot shows the loadings of frequencies on the second principal component (PC2). PC2 accounts for 1.5% of the total variance. PC1 captures the majority of the variance, representing the overall spectral profile. PC2 captures a smaller portion of the variance, likely representing more subtle spectral variations or features. The score plot (Figure 6B) illustrates the relationship between the fibers in this case, untreated, argon plasma-treated for 30 min and 4 h at 80 Hz and the principal components. Each point represents a fiber, and its position on the plot reflects its scores on the PCs. The samples appear to cluster together, suggesting that they have similar spectral characteristics. This is supported by the fact that they fall within an ellipse, indicating that they are within the 95% confidence interval. While there’s some overlap, there seems to be a slight separation between the untreated fiber sample and the two other fiber samples, which are both argon plasma-treated for 4 h and 30 min at 80 Hz power.

**Figure 6:**
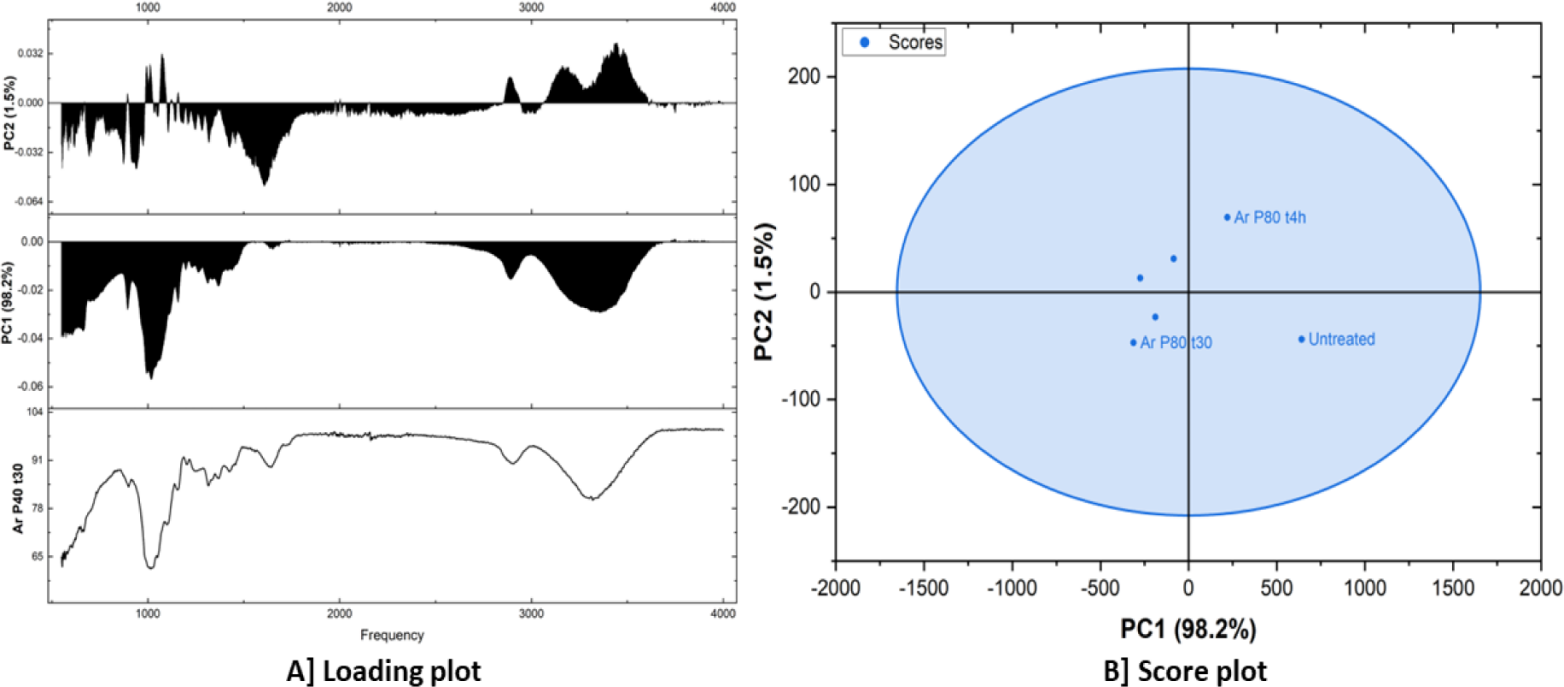
Loading plot [A] and score plot [B] obtained using PCA of the FTIR spectroscopy data.

### 3.4 Influence of Plasma Treatment on the Crystallinity of Cellulose Crystallites in Hemp Fibers

Cellulose is a semicrystalline material, comprising both an ordered crystalline structure and an amorphous region. The crystallinity index of untreated hemp fibers and argon plasma-treated fibers was analyzed using X-ray diffraction (XRD). Figure 7 presents the diffraction patterns for both untreated and plasma-treated hemp fibers. Peak fitting was performed to distinguish between the amorphous and crystalline regions. The cellulose molecule typically exhibits four characteristic peaks in the XRD pattern at approximately 2θ = 15°, 16°, 22°, and 23°.^89^ Figure 7A displays the XRD pattern of untreated hemp fibers with fitted peaks at these positions. Similarly, Figure 7B,C illustrates the XRD patterns and fitted peaks for hemp fibers treated with argon plasma at 40 and 80 Hz for 30 min. Additionally, Figure 7D,E shows the diffraction patterns for samples subjected to argon plasma treatment for 4 h at 40 and 80 Hz, respectively. Figure 7F, therefore, represents the XRD pattern comparing argon plasma-treated and untreated hemp fibers. The crystallinity index was thus determined using the peak-fitting method demonstrated in the literature review by Salem et al.^89^ Table 3 shows the crystallinity index for untreated and argon plasma-treated hemp fibers.

**Figure 7:**
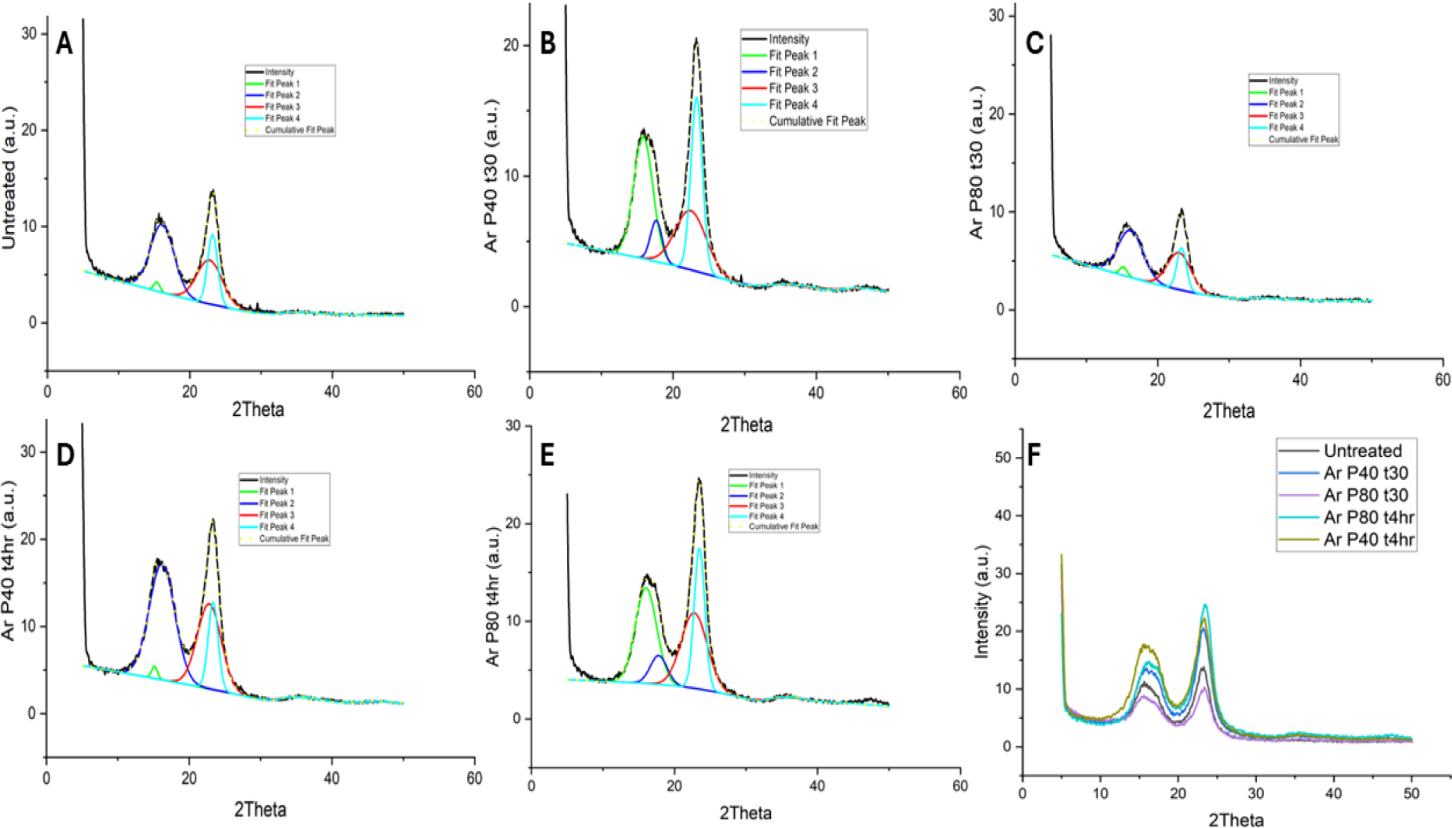
X-ray diffraction patterns along with the peak deconvolutions of A: Untreated; B: Hemp fibers argon plasma-treated for 30 min at 40 Hz power; C: Hemp fibers argon plasma-treated for 30 min at 80 Hz power; D: Hemp fibers argon plasma-treated for 4 h at 40 Hz power; E: Hemp fibers argon plasma-treated for 4 h at 80 Hz power; F: Combined X-ray diffraction graph for all the samples.

**Table 3:** Crystallinity Index of Untreated and Plasma-Treated Hemp Fibers

Sample	Crystallinity index (%)	Standard deviation (%)	Standard error (%)
Untreated	75	2.6	0.3
Ar P40 t30	63	3.0	0.5
Ar P80 t30	86	2.5	0.2
Ar P80 t4h	55	2	0.2
Ar P40 t4h	50	2.5	0.3

Interestingly, hemp fibers treated with argon plasma at 80 Hz for 30 min exhibited the highest crystallinity of 86%, whereas prolonged exposure (4 h) led to a significant reduction in crystallinity (∼50%) compared to untreated fibers (75%). Jasti and Biswas analyzed the crystallinity of hemp fibers using the peak fitting method, reporting a crystallinity range of 49% to 85%.^90^ A higher degree of surface etching was observed after 4 h of argon plasma treatment, suggesting that the removal of weakly bonded amorphous components such as lignin, pectin, or hemicellulose may facilitate the ordering of cellulose crystallites. This reorganization could contribute to sharper diffraction peaks while simultaneously decreasing the overall crystallinity of the fiber.^89^ Additionally, argon plasma treatment may induce the realignment of cellulose microfibrils along specific orientations, further enhancing peak sharpness.^89^ Fibers that underwent argon plasma treatment for 30 min at 40 Hz power exhibit a surface roughness of 300 nm, which can thus be attributed to the decrease in overall crystallinity. Das et al.^91^ observed macromolecular transitions in kapok fibers following 30 min of RF plasma treatment, attributing an ∼10% increase in crystallinity due to partial lignin removal. Although focused on a different fiber, their observations resonate with our findings, where similar structural modifications were noted post-treatment.

Crystallite size was calculated using the Debye–Scherrer equation^89^ to understand these structural changes better. Figure 8 presents the four identified crystallites along with their respective sizes in nanometers. The (002) plane, which is found at 2θ = 22°, is strongly related to cellulose crystallinity, representing the stacking of cellulose chains along the fiber axis.^92^ Referring to Figure 8, it can be observed that crystallite size has no significant change, indicating that argon plasma treatment is confined only to a surficial interaction.

**Figure 8:**
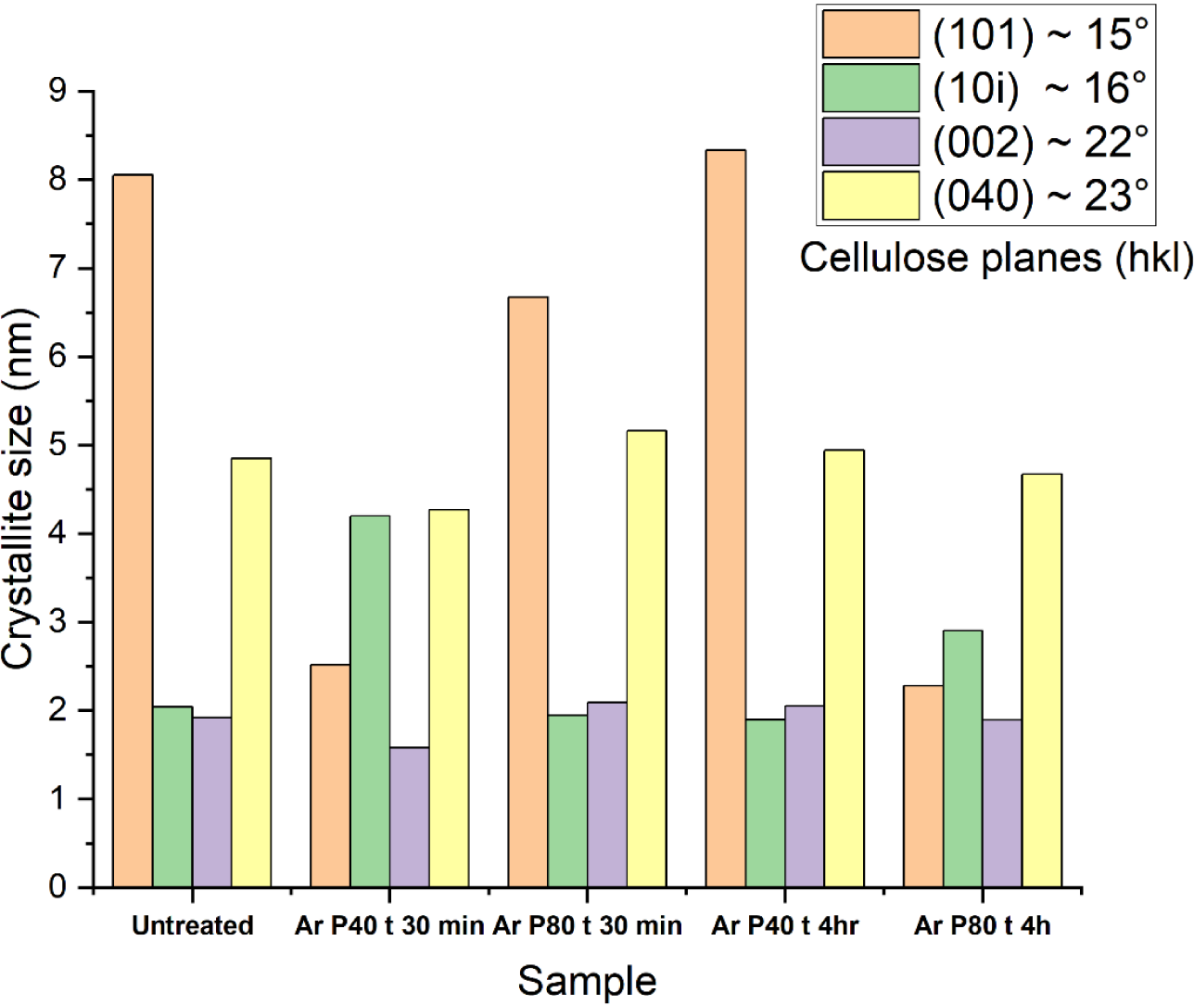
Crystallite size of untreated and argon plasma-treated fibers.

Cellulose crystals at the (101) and (101̅) planes indicate the lateral packing of cellulose chains, with a notable variation observed in fibers treated with argon plasma for 4 h at 80 Hz, suggesting localized reordering. In contrast, the (040) plane, which appears at approximately 2θ = 23°,^92^ corresponds to intermolecular hydrogen bonding within cellulose sheets. Notably, no significant changes were observed in this crystalline region, and the crystallite size remains unchanged across all the plasma treatments. This suggests that the argon plasma treatment is mainly confined to the fiber surface. As the degree of crystallinity is related to the mechanical properties of the fiber, the tensile strength of untreated and plasma-treated hemp fibers was determined.

### 3.5 Influence of Plasma Pretreatment on the Tensile Strength of Hemp Fibers

Figure 9 shows representative stress–strain results for untreated and plasma-treated hemp fibers. As shown, both argon and oxygen plasma treatments for 30 min do not affect the tensile strength of the fibers remains unaltered. It may be speculated that plasma treatment may induce realignment of the cellulose crystallites, leading to more efficient load transfer,^93^ resulting in plasma-treated fibers exhibiting a similar strength to the untreated, suggesting that argon and oxygen plasma treatments lead to minimal changes in the bulk properties of the fiber. Notably, fibers subjected to argon plasma treatment at 80 Hz for 30 min closely follow the stress–strain curve of the untreated fibers, suggesting no adverse effect on the tensile properties. Additionally, these fibers have 85% crystallinity, suggesting that the fundamental cellulose crystalline structure remained largely intact, preserving the strength loss.

**Figure 9:**
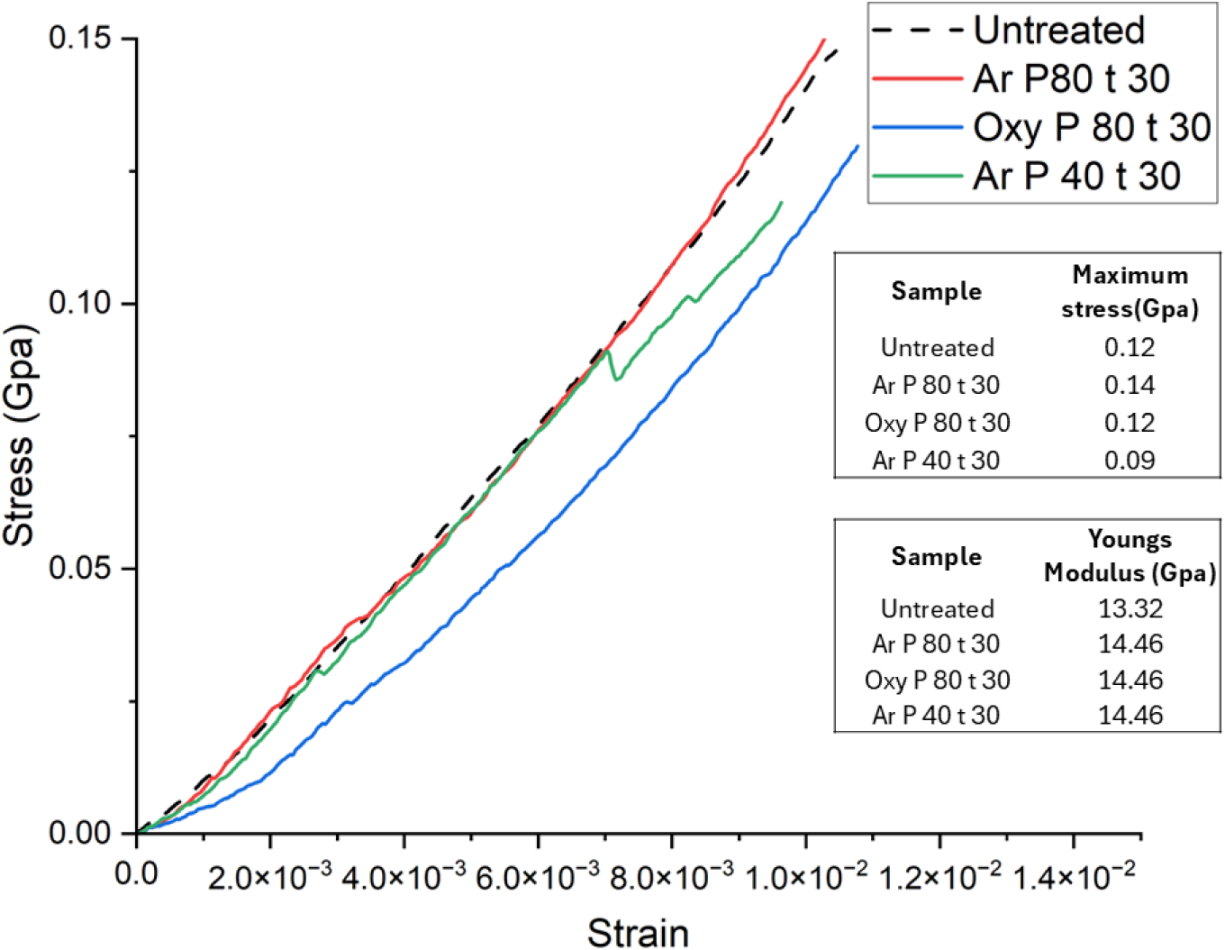
Stress–strain results for hemp fibers along with surface response analysis, where Ar P80 Hz for 30 min represents argon plasma treatment for 30 min at 80 Hz power and Oxy P80 Hz for 30 min represents oxygen plasma treatment for 30 min at 80 Hz power.

In cellulose, the (002) crystal plane plays a crucial role in governing longitudinal stress transfer. The (101) and (040) crystal planes are associated with intermolecular hydrogen bonding, along with the (101̅) plane exhibiting the largest crystallite size among all tested samples, which may play a critical role in reinforcing fiber structure, along with the (101) and (101̅) planes contributing to fiber rigidity and tensile strength. The crystallite size for the (002) plane (Figure 8) does not show a substantial difference from that of the untreated fibers, indicating that argon plasma treatment remains confined to the surface. Fibers treated with argon plasma at 40 Hz for 30 min exhibit a slightly reduced tensile strength (∼0.09 GPa) than untreated fibers (Figure 9) and have a crystallinity of 63%. Such a slight decrease in the tensile strength may be correlated with the reduced crystallite size for the (002) plane (Figure 8). These findings indicate that argon plasma treatment could enhance the strength of intermolecular hydrogen bonds, ultimately preserving the mechanical performance of hemp fibers, although advanced research is needed for concrete evidence.

Hemp fibers plasma-treated with oxygen gas for a duration of 30 min at 80 Hz power show a tensile strength (∼0.12 GPa) similar to the untreated fibers. This indicates that oxygen plasma treatment remained confined to the surface without penetrating the bulk structure of the fiber. Moreover, FTIR analysis of oxygen plasma-treated fibers confirms this, as the chemical fingerprint of cellulose remains unchanged. Similarly, in the case of argon plasma-treated fibers, although a higher surface roughness was observed at a micrometer scale, it did not alter the tensile properties. Hence, to confirm the hypothesis of lignin removal, fluorescence microscopy was performed on untreated and argon plasma-treated hemp fibers.

### 3.6 Influence of Plasma Treatment on Fluorescence of Lignin

Lignin naturally exhibits autofluorescence when excited at a wavelength of 488 nm.^40^ Therefore, autofluorescence microscopy was utilized as a confirmatory test to assess lignin removal following plasma treatment.^94^ This technique offers high sensitivity and specificity, enabling precise identification and localization of lignin within plant tissues.

A *z*-stack is a series of images captured at different focal planes along the *z*-axis (depth) in a sample. The microscope captures multiple images at incremental depths (*z*-positions). The resulting stack of images can be used to analyze the fluorescence intensity at different depths. Figure 10 presents both fluorescence and transmission images of untreated and plasma-treated fibers (treated for 4 h at 80 Hz power), along with the corresponding fluorescence intensity profile along the depth (*z*-axis). A comparison between the fluorescence image of the untreated fiber (Figure 10B) and the plasma-treated fiber (Figure 10D) reveals that the plasma-treated fibers exhibit fluorescence intensity lower than that of the untreated ones. A noticeable difference is observed in the fluorescence intensity peaks, with the plasma-treated sample exhibiting lower fluorescence intensity and a shallower peak.

**Figure 10:**
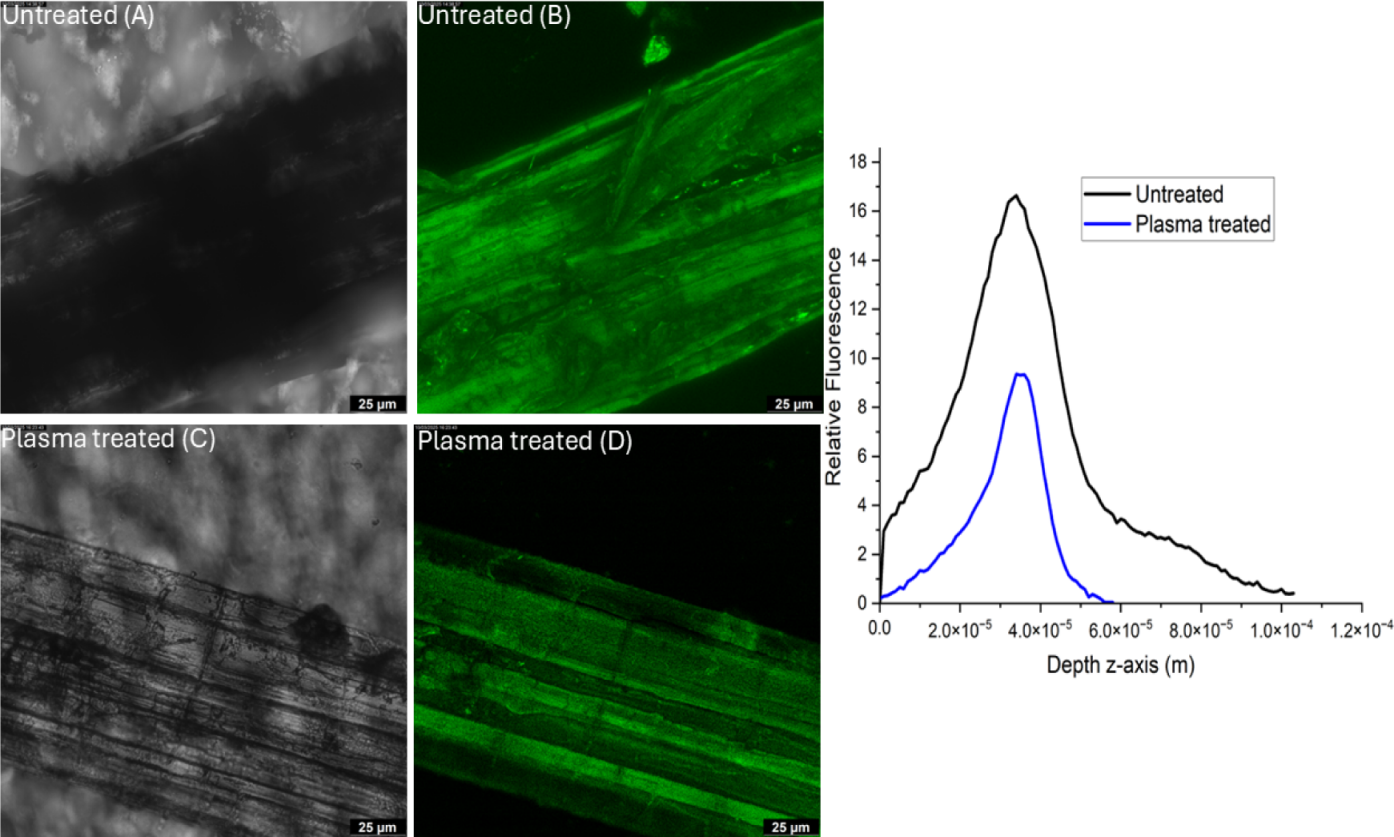
[A] Transmission image of untreated fiber, [B] autofluorescence image of untreated fiber, [C] transmission image of plasma-treated fiber, and [D] autofluorescence image of plasma-treated fiber along with relative autofluorescence intensity vs depth (*z*-axis).

The *z*-stack imaging method used in fluorescence microscopy thus confirms the hypothesis that plasma treatment removes or modifies the surface lignin.

## 4 Conclusions

In conclusion, this study highlights some physicochemical impacts of argon and oxygen plasma treatment on hemp fibers, offering a valuable approach to surface fiber modification. Results demonstrate that both the treatment duration and the type of plasma gas significantly affect the degree of surface etching, with surface roughness increasing from around 70 to approximately 299 nm, according to AFM measurements. The texturing observed on the fiber surface is attributed to surface degradation and fibrillation of cellulose, with etching probably proceeding in a layer-by-layer manner. These results highlight the potential of plasma treatment as an environmentally friendly method to enhance the functional performance of natural fibers, such as hemp. The induced surface morphological changes observed via SEM and AFM can significantly improve surface reactivity, thereby broadening the fiber’s applicability across both apparel and technical textile domains.

Autofluorescence mapping also points toward lignin loss as a result of the plasma treatment. The water contact angle of untreated hemp fibers, of about 69°, is reduced to 0° following plasma treatment with both argon and oxygen, signifying a marked increase in surface energy resulting from surface roughening. FTIR analysis of argon plasma-treated hemp fibers shows no major functional group changes, although changes to the peak envelope suggest physicochemical modifications consistent with changes to the bound-cellulose water levels. Similarly, oxygen plasma treatment indicates a possible surface functionalization without alteration in the bulk cellulose structure. X-ray diffraction analysis suggests a decrease in crystallinity after a 4 h argon plasma treatment. Analysis of crystallite suggests that plasma treatment remained confined to the fiber surface without altering the crystallite size of the fibers, with the tensile strength of the fibers remaining unchanged after 30 min of plasma treatment.

Overall, plasma treatment may prove to be a viable, low-chemical-contact technique for the manipulation of hemp fiber surface morphology, achieving surface modification with minimal water and chemical consumption while preserving the bulk properties of the fiber. These findings position plasma treatment as a sustainable and potentially impactful method for advancing the utility of natural fibers such as hemp in diverse applications such as garments and technical textiles.

## Supplementary Material



## Notes

AFM section analysis of untreated and argon plasma-treated Hemp fibers (Figure S1); AFM section analysis of argon plasma-treated Hemp fibers (Figure S2); AFM section analysis of oxygen plasma-treated Hemp fibers (Figure S3) and 4 h at 40 Hz; and surface roughness (RMS) data with surface response analysis (Figure S4) (PDF)
