# Corrections to “Study of Hemp Fiber Properties Modified via Long-Duration Low-Pressure Argon and Oxygen Plasma Treatments”

**DOI:** 10.1021/acsomega.6c01178

**Published:** 2026-03-10

**Authors:** Kunal Bapat, Lekshmi Kailas, Christopher Pask, Terence P. Kee, Stephen Russell

**Description of correction:** The ACS Omega editorial team made some typographical errors in the units of the AFM scans, which jeopardize the logic. For instance, changing micrometre (μm) to millimeter (mm).

**The mistakes made by the journal editorial team are pointed out below:**

1. Page 61437, lines 251–252: Three different areas on three different fibers were imaged, and scan sizes at 5, 10, 20, and 30 mm were collected.
2. Page 61440, Figure 3 caption: AFM (10 mm × 10 μm)
3. Page 61441, lines 495–496: Supporting Information (Figures S1–S3) determined for a 10 nm section in the AFM scan.
4. Page 61441, lines 540–541: time of 30 min produced a surface roughness of 182 nm for a 10 mm × 10 mm scan

**We would like to make the following corrections in the units, as all AFM scans were done in μm:**

1. Page 61437, lines 251–252: Three different areas on three different fibers were imaged, and scan sizes at 5, 10, 20, and 30 μm mm were collected.
2. Page 61440, Figure 3 caption: AFM (10 μm mm × 10 μm)
3. Page 61441, lines 495–496: Supporting Information (Figures S1–S3) determined for a 10 μm nm section in the AFM scan.
4. Page 61441, lines 540–541: time of 30 min produced a surface roughness of 182 nm for a 10 μm mm  × 10 μm mm scan

