# Supplementary material for: Study of Hemp Fiber Properties Modified via Long-Duration Low-Pressure Argon and Oxygen Plasma Treatments
Source: ACS Omega. 2025 Dec 11;10(50):61435–51. doi: 10.1021/acsomega.5c06420 (PMC12750193; doi:10.1021/acsomega.5c06420)
Supplement: Supplementary file 1 [file ao5c06420_si_001.pdf]

Supporting Information (SI)

For

Study of hemp fiber properties modified via long duration low-pressure argon and oxygen plasma treatments

Kunal Bapat [1], Lekshmi Kailas [2], Christopher Pask [1], Terence Kee* [1], Stephen Russell [3]

[1] *School of Chemistry, University of Leeds, Woodhouse Lane, LS2 9JT, UK*

[2] *School of Physics and Astronomy, University of Leeds, Woodhouse Lane, LS2 9JT, UK*

[3] *Leeds Institute of Textiles and Colour, School of Design, University of Leeds, Woodhouse Lane, LS2 9JT, UK*

1.1 Section analysis of AFM scans

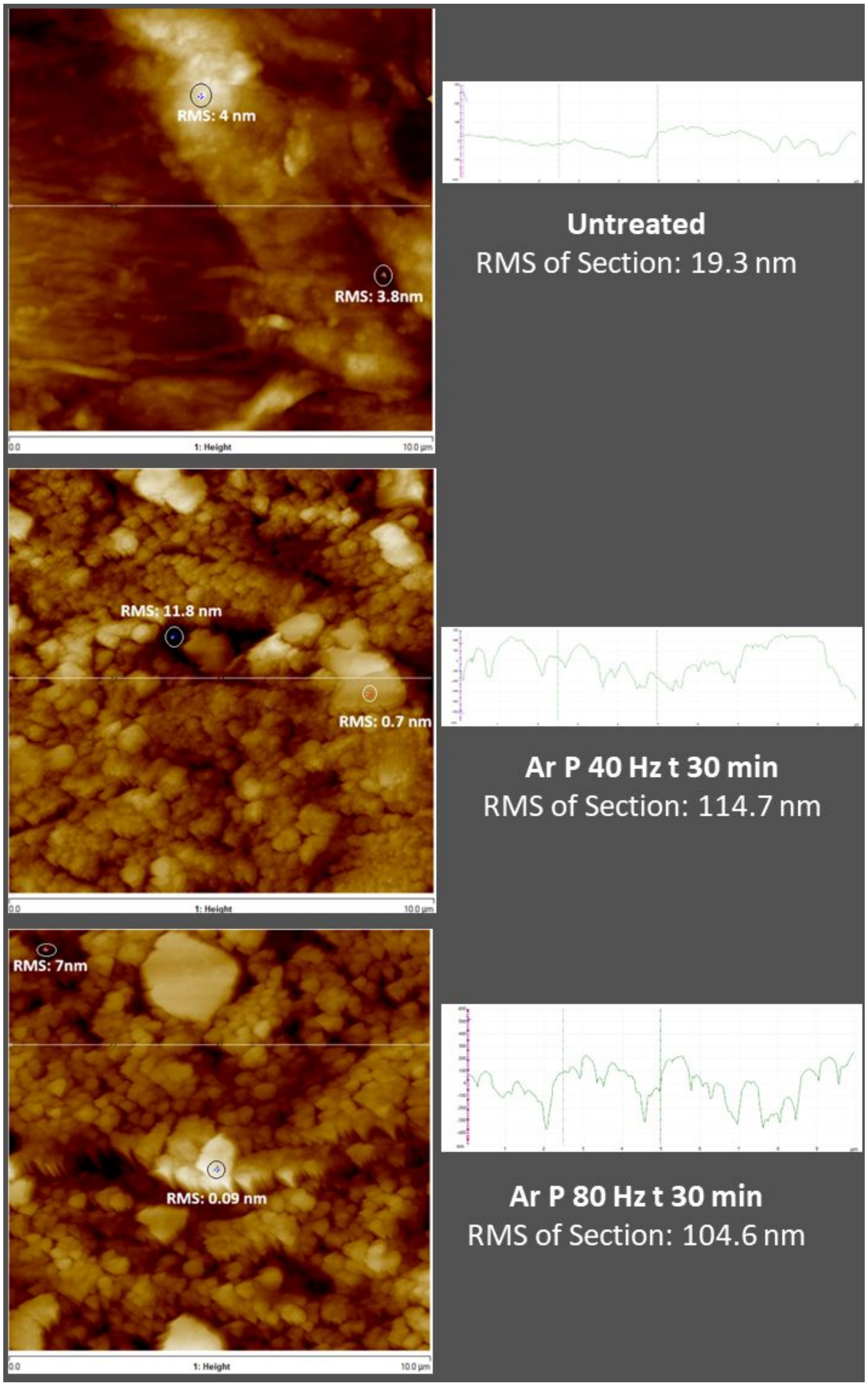

Figure S1 AFM section analysis of untreated and argon plasma treated hemp fibres for 30 min at 40 and 80 Hz power

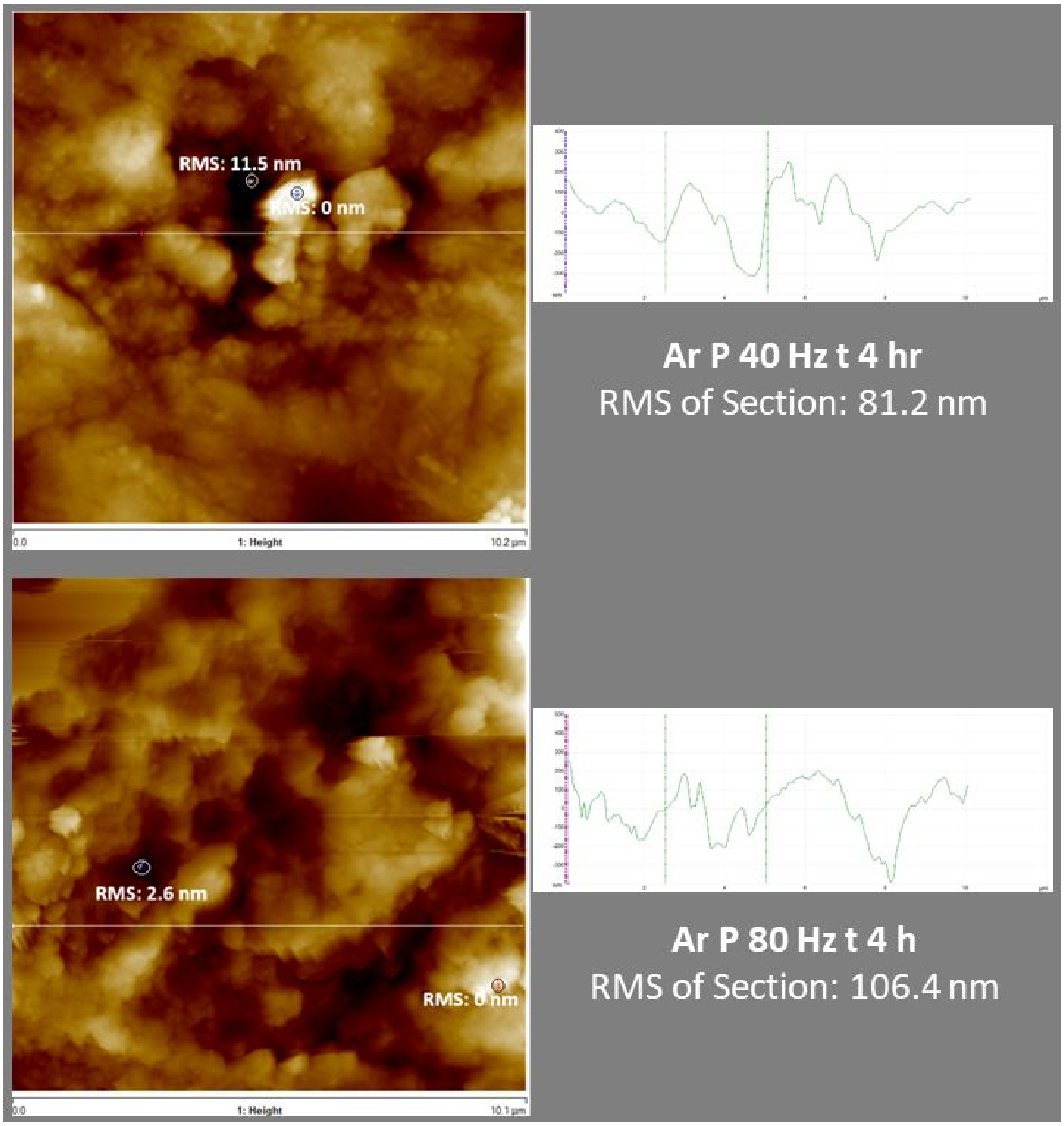

Figure S2 AFM section analysis of argon plasma treated hemp fibres for 4 hr at 40 and 80 Hz power

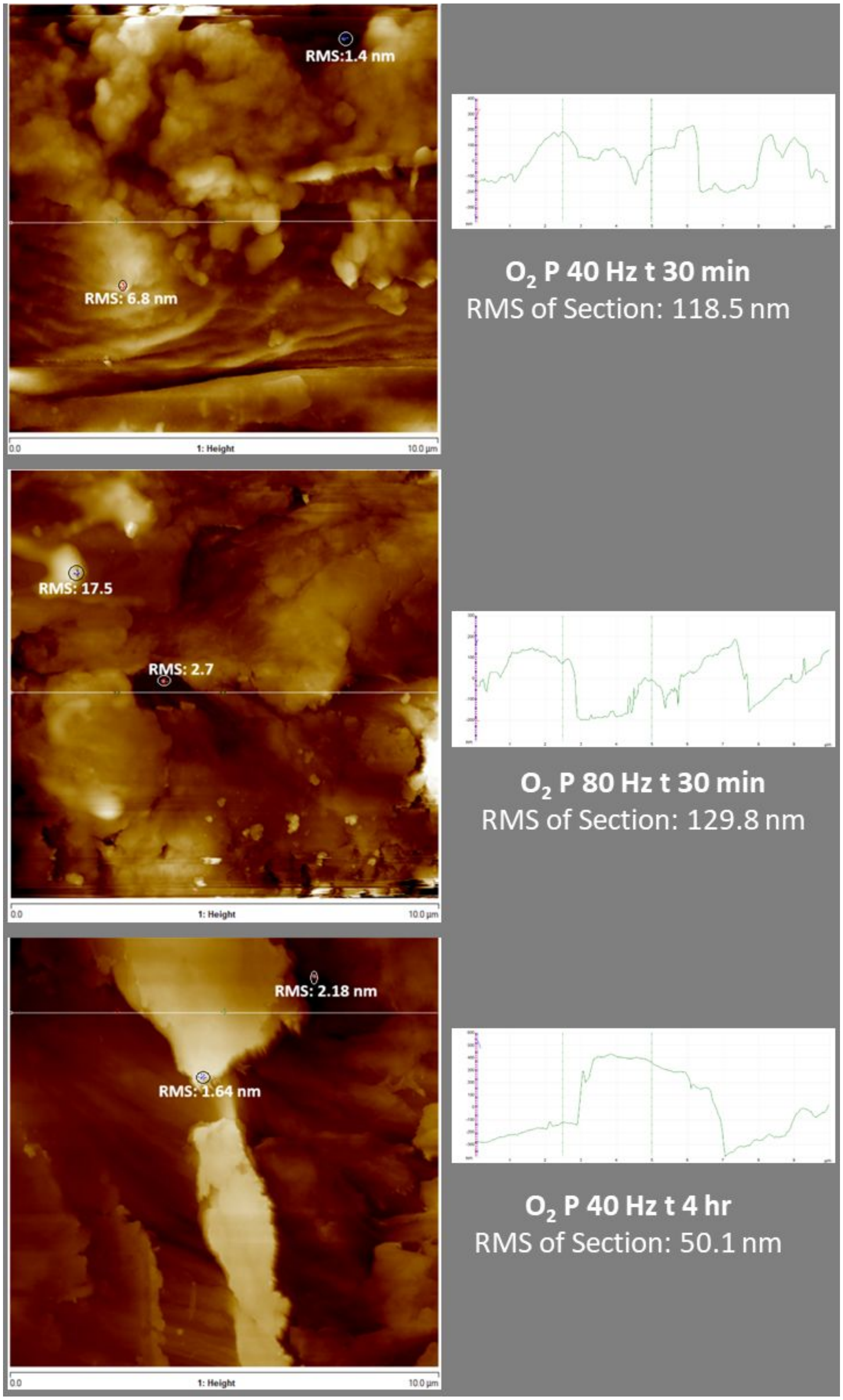

Figure S3 AFM section analysis of oxygen plasma treated hemp fibres for 30 min at 40 and 80 Hz power along with 4 h oxygen plasma treated fibres at 40 Hz power

1.2 Statistical analysis of fibre-surface roughness

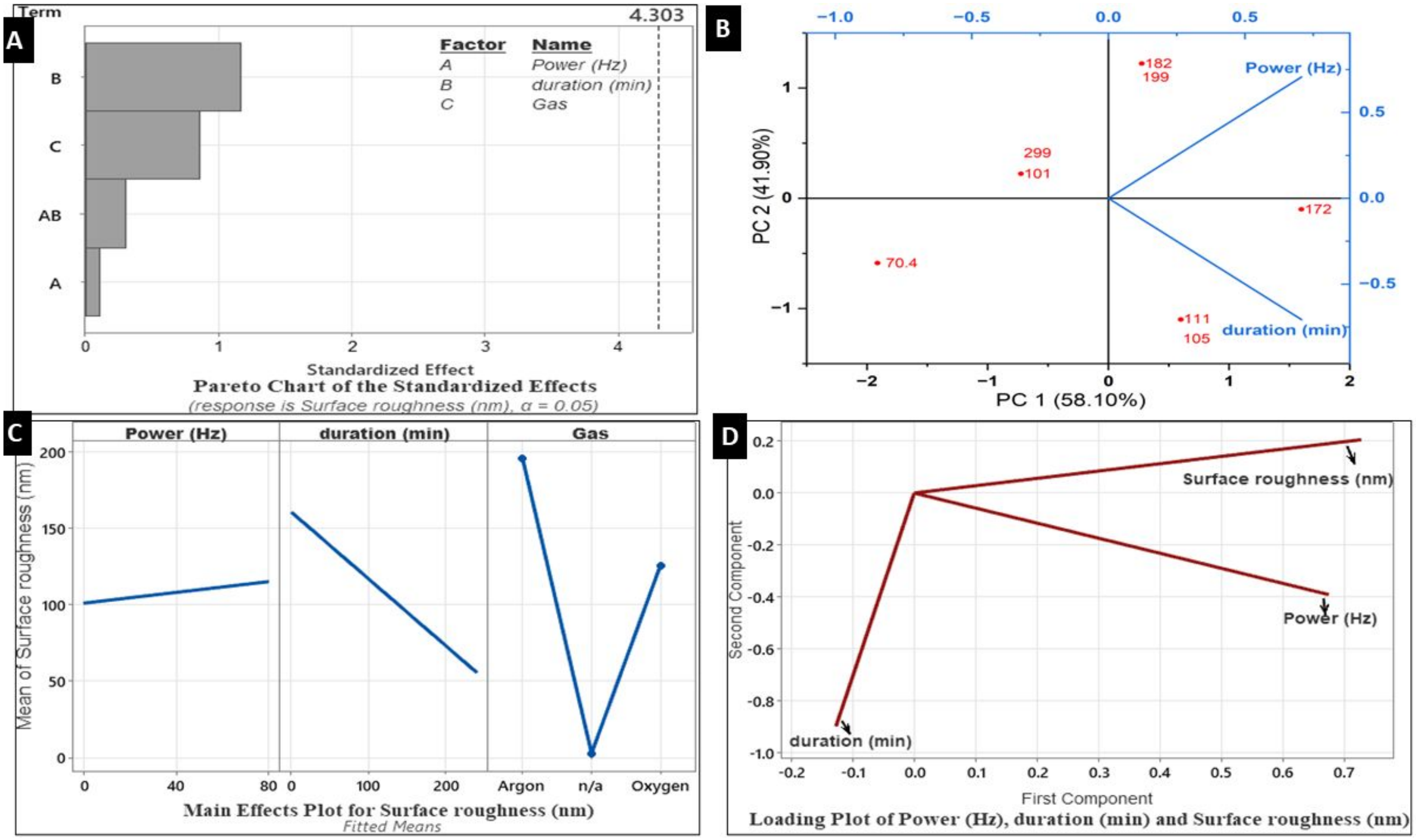

Figure S4 Surface roughness (RMS) of hemp fibres along with surface response analysis wherein A: Pareto chart of the effects of factors, B: Principal component analysis of plasma treatment factors (gas, power and treatment time) contributing towards surface roughness, C: Main effects plot for surface roughness and D: Loading plot of plasma treatment factors (power and treatment time) and surface roughness

Figure S4 presents the statistical analysis for the surface roughness (RMS) data derived from AFM scans of untreated and plasma-treated fibres, incorporating surface response analysis and multivariate analysis to evaluate the influence of various treatment parameters. Figure S4 A shows a Pareto chart of standardised effects, where factors influencing surface roughness (nm) are analysed. The factors include Power (Hz) [A], Duration (min) [B], and Gas type [C]. The chart reveals that Power (Hz) has the strongest influence on surface roughness, aligning with the principal component, PC 1, which accounts for 58.10% of the variance, as confirmed in Figure S4 B. The PCA analysis suggests that higher power settings may result in greater surface roughness. Additionally, duration of plasma treatment is the second most significant factor, associated with

PC 2, accounting for 41.90% of the variance. Interestingly, plasma treatment for shorter durations of about 30 min corresponds to higher roughness, while longer durations of 240 min may lead to smoother surfaces, indicating an inverse relationship. Among gases, plasma generated using argon gas consistently leads to higher surface roughness compared to oxygen gas under similar conditions. For instance, hemp fibres plasma treated at 40 Hz power for 30 min with argon gas result in a surface roughness of 299 nm, while fibres treated with the same conditions but using oxygen gas result in a fibre having 101 nm of surface roughness. Notably, high power (80 Hz) combined with short duration (30 min) plasma treatment results in high surface roughness ~199 nm for argon gas plasma and ~182 nm for oxygen gas plasma. In contrast, low power (40 Hz) with extended duration (240 min) yields lower roughness, ~111 nm for oxygen gas plasma, ~105 nm for argon gas plasma. This highlights that extended treatment duration can mitigate the roughness induced by higher power levels. Thus, referring to Figure S4 B, it can be concluded that Power (Hz) and surface roughness are closely aligned along PC 1, demonstrating a positive correlation. Duration (min) aligns oppositely along PC 2, showing a negative correlation with surface roughness, which aligns well with the previous interpretation. Figure S4 C shows the main effects plot, where the influence of individual factors on surface roughness is shown. The Duration panel illustrates a declining trend, which suggests that longer treatment leads to smoother surfaces. The Gas panel emphasises significant differences in roughness depending on gas type, with argon gas plasma leading to the roughest textures and oxygen gas plasma resulting in moderately rougher surfaces compared to untreated fibre. The Power panel shows an upward slope, indicating that higher power increases surface roughness, albeit to a lesser extent compared to gas and duration. Figure S4 D shows the PCA loading plot, where the vector for surface roughness closely aligns with Power (Hz), indicating a strong positive relationship. In contrast, the vector for duration points in the opposite direction, suggesting a strong negative correlation with surface roughness. The angle between the power and duration vectors suggests a weak correlation between these two parameters. These relationships reinforce earlier findings that surface roughness increases with power but decreases with treatment duration. Therefore, the statistical analysis suggests that plasma treatment operates as a synergistic process, where gas type, power, and treatment duration collectively influence surface modification. While each factor holds individual significance, it is the optimal combination of all three parameters that yields the most effective surface roughness outcomes.
